# First-class – biosynthesis of 6-MSA and bostrycoidin type I polyketides in *Yarrowia lipolytica*

**DOI:** 10.3389/ffunb.2024.1327777

**Published:** 2024-03-22

**Authors:** Mihaela Bejenari, Eva Mie Lang Spedtsberg, Julie Mathiesen, Alexandra Claire Jeppesen, Lucia Cernat, Aouregane Toussaint, Cristina Apostol, Victor Stoianov, Tobias Bruun Pedersen, Mikkel Rank Nielsen, Jens Laurids Sørensen

**Affiliations:** ^1^ Department of Chemistry and Bioscience, Aalborg University, Esbjerg, Denmark; ^2^ Department of Energy, Aalborg University, Esbjerg, Denmark; ^3^ Université Grenoble Alpes, Laboratoire de Physiologie Cellulaire Végétale, CEA, CNRS, INRA, IRIG-LPCV, Grenoble, France

**Keywords:** heterologous production, pigment, yeast, polyketide synthase (PKS), *Fusarium*, fusarubin, 6-MSA, *Yarrowia lipolytica*

## Abstract

Fungal polyketides are a large group of secondary metabolites, valuable due to their diverse spectrum of pharmacological activities. Polyketide biosynthesis in filamentous fungi presents some challenges: small yield and low-purity titers. To tackle these issues, we switched to the yeast *Yarrowia lipolytica*, an easily cultivable heterologous host. As an oleaginous yeast, *Y. lipolytica* displays a high flux of acetyl- and malonyl-CoA precursors used in lipid synthesis. Likewise, acetyl- and malonyl-CoA are the building blocks of many natural polyketides, and we explored the possibility of redirecting this flux toward polyketide production. Despite its promising prospect, *Y. lipolytica* has so far only been used for heterologous expression of simple type III polyketide synthases (PKSs) from plants. Therefore, we decided to evaluate the potential of *Y. lipolytica* by targeting the more complex fungal polyketides synthesized by type I PKSs. We employed a CRISPR-Cas9-mediated genome editing method to achieve markerless gene integration of the genes responsible for bostrycoidin biosynthesis in Fusarium solani (*fsr1*, *fsr2*, and *fsr3*) and 6-methylsalicylic acid (6-MSA) biosynthesis in Aspergillus hancockii (6MSAS). Moreover, we attempted titer optimization through metabolic engineering by overexpressing two enzymes, TGL4 and AOX2, involved in lipid β-oxidation, but we did not observe an effect on polyketide production. With maximum titers of 403 mg/L 6-MSA and 35 mg/L bostrycoidin, the latter being substantially higher than our previous results in *Saccharomyces cerevisiae* (2.2 mg/L), this work demonstrates the potential of *Y. lipolytica* as a platform for heterologous production of complex fungal polyketides.

## 1 Introduction

Polyketides represent a large group of natural products with immense structural variation despite originating from the same simple acyl precursors: acetyl- and malonyl-CoA (Staunton and Weissman, 2001; Hertweck, 2009). The precursor subunits are assembled through condensation reactions under the catalysis of polyketide synthases (PKSs), categorized into three types (I, II, and III). Type I PKSs are large, iterative, multidomain enzymes consisting of three characteristic core domains – β-ketosynthase (KS), acyltransferase (AT), and acyl-carrier protein (ACP) – along with additional domains responsible for reduction, methylation, and product release (McDaniel et al., 1994). By comparison, type II PKSs are formed as aggregates of discrete proteins and represent non-iterative, large enzyme complexes (Hertweck et al., 2007). The simplest PKSs are the type III polyketide synthases, which are homodimers of KS domains (Shimizu et al., 2017). The structural diversity of polyketides is also reflected in their biological effects, which can vary from potent carcinogenic compounds, such as aflatoxin B1 (Rushing and Selim, 2019), to pharmaceutical cholesterol-lowering drugs, such as lovastatin (Tobert, 2003). These bioactive properties drive the scientific community’s interest in polyketide biosynthesis, and heterologous hosts have become a tool for polyketide discovery and production optimization (Alberti et al., 2017; Kjærbølling et al., 2019). Due to an extensive molecular toolbox (Harvey et al., 2018) and a lack of interfering non-target metabolites (Bond et al., 2016), baker’s yeast *Saccharomyces cerevisiae* became a popular choice for heterologous production. However, the potential of *S. cerevisiae* as a host for the heterologous polyketide production is limited by the low flux of acetyl- and malonyl-CoA, with optimization of metabolic fluxes being necessary to obtain higher yields (Wattanachaisaereekul et al., 2008; Chen et al., 2013; Lian et al., 2014; Cardenas and Da Silva, 2016; Tippelt and Nett, 2021).

In the search for more potent yeasts with higher polyketide production prospects, *Yarrowia lipolytica* has emerged as one of the most promising species. This dimorphic oleaginous yeast can use a wide array of carbon sources, is naturally efficient at protein secretion, but most notably, has high acetyl- and malonyl-CoA fluxes natively used by fatty acid synthases to produce lipids at levels exceeding 50% of the dry cell weight (Papanikolaou and Aggelis, 2002; André et al., 2009; Beopoulos et al., 2009; Barth and Gaillardin, 1997). Moreover, *Y. lipolytica* has a well-equipped molecular toolbox (Bredeweg et al., 2017; Markham and Alper, 2018), which includes methods for targeted gene integration and multicomponent DNA assembly (Celińska et al., 2017; Wong et al., 2017; Holkenbrink et al., 2018), as well as a suite of native or engineered promoters (Madzak et al., 1999; Madzak et al., 2000; Blazeck et al., 2011; Blazeck et al., 2013; Schwartz et al., 2016; Shabbir Hussain et al., 2016; Xiong and Chen, 2020) and terminators (Curran et al., 2015). The comprehensive toolbox enables efficient genetic engineering of the yeast. Specifically, genes can be integrated into the genome with, e.g., CRISPR-Cas9-based systems, while the promoters and terminators allow inducible, repressible, and constitutive expression of these genes in a tunable manner (Wong et al., 2017; Holkenbrink et al., 2018; Schwartz et al., 2019).

Several reviews have hailed the capability of *Y. lipolytica* to produce polyketides upon rewiring the acetyl- and malonyl-CoA flow from lipid biosynthesis [e.g (Muhammad et al., 2020; Miller and Alper, 2019; Ma et al., 2020)], and a few type III PKS products have already been heterologously synthesized in this host (**Table 1**). The structurally simple triacetic acid lactone (TAL; C_6_H_6_O_3_) was the first polyketide produced in *Y. lipolytica* by inserting four copies of the responsible PKS gene (*g2ps1*). The initial 2.1 g/L titers from tube fermentations were further enhanced through genetic optimization of the metabolic fluxes and cultivation in bioreactors, leading to a yield of almost 36 g/L (Markham et al., 2018). Other studies have also evaluated the capacity of metabolically engineered *Y. lipolytica* to produce TAL, and yields of 2.6 g/L (Yu et al., 2018) and 4.8 g/L (Liu et al., 2019b) were obtained. Additionally, *Y. lipolytica* has been genetically engineered to use other acyl substrates, such as 4-coumaroyl-CoA, and the strain was subsequently used to produce naringenin, resveratrol, and bisdemethoxycurcumin (Lv et al., 2019; Palmer et al., 2020; Sáez-Sáez et al., 2020). The resulting yields of 898 mg/L naringenin (Palmer et al., 2020) and 12.4 g/L resveratrol (Sáez-Sáez et al., 2020) are the highest titers of these compounds obtained in any species, demonstrating the potential of *Y. lipolytica* as a polyketide producer.

**Table 1:** Overview of heterologous production of polyketide-derived compounds in *Y. lipolytica*.

PKS type	Polyketide product	Titer	Reference
**Type III**	Triacetic acid lactone (TAL)	36 g/L	Markham et al. (2018)
		2.6 g/L	Yu et al. (2018)
		4.8 g/L	Liu et al. (2019b)
	Naringenin	254.2 mg/L	Lv et al. (2019)
		898 mg/L	Palmer et al. (2020)
	Resveratrol	48.7 mg/L	Palmer et al. (2020)
		12.4 g/L	Sáez-Sáez et al. (2020)
	Bisdemethoxycurcumin	0.17 mg/L	Palmer et al. (2020)
**Type I**	6-methylsalicylic acid (6-MSA)	403 mg/L	This study
	Bostrycoidin	35 mg/L	This study

**Figure 1:**
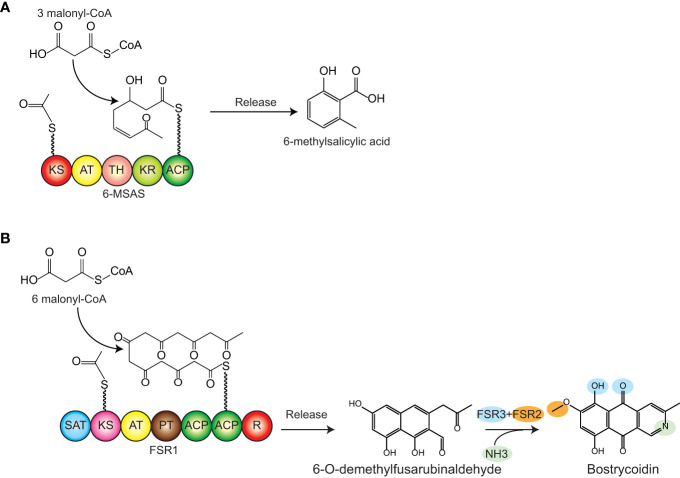
Biosynthetic pathway of 6MSA and bostrycoidin. **(A)** 6MSA is synthesized by the PKS 6-MSA synthase (6-MSAS) from one acetyl-CoA and three malonyl-CoA units. **(B)** Bostrycoidin is synthesized from one acetyl-CoA and six malonyl-CoA units. The PKS FSR1 assembles the units into 6-O-demethylfusarubinaldehyde, and following the release of this intermediary product, incorporation of ammonia (non-enzymatically), oxygenation, and o-methylation by FSR3 and FSR2, respectively, yield bostrycoidin. Domain names: SAT, starter acyl transferase; KS, ketosynthase; AT, acyl transferase; PT, product template; ACP, acyl-carrier protein; TH, thioesterase; KR, ketoreductase; R, reduction domain for product release.

To date, the reported research has focused on type III PKSs in *Y. lipolytica*, whereas the more complex type I PKSs have yet to be expressed heterologously in this host. To expand the polyketide production range in *Y. lipolytica*, we targeted two polyketides, 6-methylsalicylic acid (6-MSA), a model polyketide produced by a wide range of fungal species, and bostrycoidin, a polyketide naturally synthesized by the fungal genus *Fusarium*. 6-MSA is assembled from the acetyl- and malonyl-CoA building blocks by a 6-MSA synthase (6-MSAS), (**Figure 1A**) and has become a hub for a multitude of complex secondary metabolites, among which patulin, aculin, and terreic acid (Bejenari et al., 2023). Structurally simpler, 6-MSA was one of the first polyketides produced in heterologous hosts such as *Streptomyces coelicolor* (Bedford et al., 1995), *Escherichia coli*, and *Saccharomyces cerevisiae* (Kealey et al., 1998; Wattanachaisaereekul et al., 2007). As for the second polyketide, bostrycoidin, the gene cluster responsible for its biosynthesis is one of the three PKS clusters found in all genomically sequenced *Fusarium* species (Brown et al., 2012; Brown and Proctor, 2016; Nielsen et al., 2019). Besides the PKS (encoded by *fsr1*), the gene cluster contains an O-methyltransferase (FSR2, encoded by *fsr2*) and an FAD-dependent monooxygenase (FSR3, encoded by *fsr3*), (**Figure 1B**) (Brown et al., 2012; Studt et al., 2012; Frandsen et al., 2016). In a previous study, we used *S. cerevisiae* for heterologous production of bostrycoidin based on a galactose-inducible system, which yielded 2.2 mg/L (Pedersen et al., 2020). In this work, we hypothesized that the yields could significantly be improved by switching to *Y. lipolytica* as a production host for the type I fungal polyketides 6-MSA and bostrycoidin.

## 2 Materials and methods

### 2.1 Strains, media, and growth conditions

*Yarrowia lipolytica* strain PO1f (MatA, ura3-302, leu2-270, xpr2-322, axp-2), ATCC MYA-2613 (Madzak et al., 2000) was used as parental strain for inserting the 6-MSAS encoding gene, bostrycoidin gene cluster, together with the co-activating PPTase. Subsequently, the bostrycoidin-producing strain was used as a parental strain for ß-oxidation overexpression. *Y. lipolytica* strains were cultivated in YPD medium (1% Bacto yeast extract, 2% Bacto peptone, 2% glucose; for growth in shake flasks, glucose concentration was increased to 10%). YPD broth with 1 mg/mL of 5-FOA (Sigma Aldrich, St. Louis, MO, USA) was used for the curation from the URA3-containing donor plasmid. Transformants were selected on SD medium without leucine and uracil (0.67% Yeast nitrogen base without amino acids, 0.139% Yeast synthetic drop-out medium without histidine, leucine, tryptophan, and uracil, supplemented with histidine and tryptophan (all components from Sigma Aldrich, St. Louis, MO, USA), 2% glucose). The cultures were grown at 30°C and 200 rpm in flat and baffled bottom shake flasks.

*Escherichia coli* NEB5α high-efficiency competent cells (New England BioLabs, Ipswich, MA, USA) were used for plasmid assembly, proliferation, and maintenance. The cells were grown at 37°C, 250 rpm, in SOC medium (New England BioLabs, Ipswich, MA, USA) for recovery after transformation and in LB medium (Lennox, Sigma Aldrich, St. Louis, MO, USA) with 100 mg/L ampicillin (Amp, VWR Chemicals, Sanborn, NY, USA) for plasmid selection and propagation.

### 2.2 Plasmids and oligonucleotides

CRISPR-Cas9 plasmids used throughout this study were a gift from Ian Wheeldon (Schwartz et al., 2016; Schwartz et al., 2017). The plasmids can be accessed on Addgene through their respective accession numbers: pCRISPRyl (#70007), pCRISPRyl_A08 (#84610), pCRISPRyl_D17 (#84611), pCRISPRyl_AXP (#84608), pCRISPRyl_XPR2 (#84609), pHR_A08::*hrGFP* (#84615), pHR_D17::*hrGFP* (#84616), pHR_AXP::*hrGFP* (#84613), pHR_XPR2::*hrGFP* (#84614). The donor plasmids will be referred to as pA08, pD17, pAXP, and pXPR2.

All genes were codon optimized for *Y. lipolytica* with Gene Optimizer (Thermo Fisher Scientific, Waltham, MA, USA) (Raab et al., 2010) and ordered from GenScript (GenScript Biotech, NJ, USA). Oligonucleotides for cloning the donor vectors, for genome screening and amplification, were designed with the Primer3Plus software (Untergasser et al., 2012). Oligonucleotides for cloning sgRNA sequences were designed based on two publicly available scoring algorithms (Doench et al., 2016; Hsu et al., 2013), integrated into the Benchling software, using the Po1f genome of *Y. lipolytica*. All oligonucleotides were purchased from Eurofins Genomics (Ebersberg, Germany) and are listed in the **Supplementary Material (Supplementary Table 1)**, **Supplementary Material (Supplementary Table 2)**. The sgRNA sequences corresponding to the A08, D17, AXP, and XPR2, designed by Schwartz et al., 2017 (Schwartz et al., 2017), are indicated in **Supplementary Table 3 of Supplementary Material**.

### 2.3 Cloning of the 6-MSA and bostrycoidin genes

The codon-optimized sequences of *fsr1-3* and the helper enzyme PPTase from *Fusarium solani (FsPTT1)*, as well as *6MSAS* from *Aspergillus hancockii*, were amplified by PCR using primers with 30 bp extension tails for insertion into the expression cassette between the UAS1B8-TEF (136) promoter and CYC1 terminator of the homologous donor plasmids. All the final plasmids throughout this study were generated through Gibson Assembly, using a Gibson assembly kit (New England BioLabs, Ipswich, MA, USA) (**Supplementary Materials, Supplementary Figure 1**).

### 2.4 Cloning procedures for manipulation of ß-oxidation

Cas9 plasmids, targeting the immediate upstream location of the *Tgl4* and *POX2* genes, were assembled using the pCRISPRyl plasmid and the oligonucleotides encoding the corresponding designed sgRNAs, as per Schwartz et al., 2018 (Schwartz and Wheeldon, 2018). The pAXP::hrGFP plasmid was modified through PCR by adding a unique restriction site (RS), BglII, downstream the homologous region 2 (HR2) and removing the *hrGFP* gene and CYC1 terminator (**Supplementary Material, Supplementary Figure 2A**). The newly modified plasmid permitted facile removal and replacement of HR1 via restriction enzyme digestion with KspAI and BcuI and HR2 with AvrII and BglII. Subsequent gel purification of digested plasmid and Gibson Assembly with PCR amplified donor regions resulted in the final plasmids consisting of the new homology arms and the UAS1B8-TEF(136) promoter, ready for subsequent use in overexpression (OE) experiments. The HRs were designed based on the Po1f genome of *Y. lipolytica*, and the sgRNAs were selected such that the PAM sequence and a few additional base pairs would be removed after genomic integration. The HRs were PCR amplified, and Gibson Assembly was performed iteratively for each 1kb homology arm (**Supplementary Material, Supplementary Figure 2B**).

### 2.5 Transformation and validation of *Y. lipolytica*

Transformation of *Y. lipolytica* was performed according to Schwartz et al., 2018 (Schwartz and Wheeldon, 2018) with the following modifications: 1) 500 μL stationary phase cells were aliquoted to microcentrifuge tubes; 2) Sonicated Salmon Sperm DNA (ssDNA, Abnova, Taipei, Taiwan) was denatured at 100°C for 5 minutes and used at a working concentration of 10 mg/mL; hence only 2.4 μL were used to achieve the desired concentration of 24 μg; 3) the amount of plasmid DNA was aimed at 1 μg of each, pCRISPRyl and donor plasmid, with a maximum total volume of 10 µL DNA; 4) the total volume of the transformation mix was 278, therefore depending on the volume of used DNA, water could be added up to 12.8 μL; 5) instead of 15 μL triacetin mix, 12.8 μL of 1M DTT (Thermo Fisher Scientific, Waltham, MA, USA) was used; 6) the final 100 μL of transformed cells were plated on SD-Leu-Ura plates.

To obtain the *YL::fsr1/3* strain, genes from the bostrycoidin gene cluster were integrated in a successive manner where *fsr1*, *fsr2, fsr3*, and *FsPPT1* were integrated into the A08, XPR2, AXP, and D17 sites, respectively, through CRISPR-Cas9 mediated transformation guided by the associated pCRISPRyl guide plasmids (**Supplementary Material, Supplementary Figure 3**). Similarly, the *YL::6MSAS* strain was obtained by integrating *6-MSAS* into the A08 locus of *YL::FsPPT1*, a *Y. lipolytica* strain already containing *FsPPT1* in the D17 locus. Subsequently, *YL::fsr1/3* was transformed to overexpress the *Tgl4* and *POX2* genes, alone and combined. After each round, the resulting transformants were curated with 5-FOA and validated by diagnostic colony PCR (cPCR) (Schwartz and Wheeldon, 2018).

Strain screening and validation were conducted with a two-primer cPCR for the *YL::fsr1/3* strain and a three-primer cPCR for the *YL::6MSAS* and *Tgl4/POX2* OE strains. A fresh colony was picked with an inoculation loop, and after streaking on a YPD plate for library generation, the leftover cells were added directly to the PCR mix containing 0.5 μM of each primer. The denaturation time for the first PCR step was adjusted to 10 min to ensure cell wall disruption and DNA release in the mix. Additionally, after the transfer of the entire bostrycoidin gene cluster, validation of *YL::fsr1/3 strain* was performed via sequencing using a R9.4.1 MinION Flow Cell (Oxford Nanopore Technologies, ONT, Oxford, United Kingdom) according to the SQK-LSK109 protocol from ONT. DNA extraction and purification were performed according to Petersen et al., 2022 (Petersen et al., 2022) except for the removal of small fragments step, which was skipped. Genome assembly, relative to the reference genome of *Y. lipolytica*, and visualization of mapped reads were performed in CLC Genomics Workbench version 22.0 (QIAGEN, Aarhus, Denmark).

### 2.6 Chemical analyses

Starter cultures of the *Y. lipolytica* strains for shake flask fermentations were inoculated from single colonies and grown for 24 h. After measuring OD_600_ on a NanoDrop 2000c spectrophotometer (Thermo Fisher Scientific, Waltham, MA, USA), 50 mL of YPD (10% glucose) were inoculated with approximately the same number of cells from the starter cultures and grown for seven days. For bostrycoidin and 6-MSA analysis, samples were prepared by centrifugation at 12,100 x g for 1 min and mixing 400 μL supernatant with 100 μL methanol. For glucose analysis, the samples were centrifuged at 12,100 x g for 1 min, and 500 μL supernatant was filtered through 0.45 μm Nylon syringe filters (Frisenette, Knebel, Denmark) directly into 2 mL HPLC vials.

Bostrycoidin and 6-MSA analysis was performed on a Thermo Vantage triple stage quadrupole mass spectrometer (Thermo Fisher Scientific, San Joseí, CA, USA) with a heated electrospray ionization probe operating in negative mode. The sample was injected and separated on a C6-Phenyl column (Gemini, 3 μm x 50 mm x 2 mm, 110 A°C pore size, Phenomenex, Torrance, CA, USA) by using a constant flow of 0.4 mL/min and applying a linear gradient of acetonitrile and water, both containing 0.1% formic acid. The gradient initiated at 5% acetonitrile, increasing to 100% over 10 min and being held at 100% for 3 min.

Glucose was quantified on an HPLC-RID system (Perkin Elmer Series 200, Perkin Elmer, MA, USA) fitted with an Aminex HPX-87H ion exclusion column (300 x 7.8 mm, Bio-Rad Laboratories, Hercules, CA, USA) and a Perkin Elmer series 200a Refractive index detector. The column oven was held at 65°C with 5 mM sulfuric acid as the mobile phase, and the samples were analyzed using a constant flow of 0.8 mL/min for 20 min. The calibration was accomplished using glucose within the 1 - 100 g/L range.

## 3 Results

### 3.1 Generation of *Y. lipolytica* strains capable of producing fungal polyketides

As an oleaginous yeast, *Y. lipolytica* appears to be a promising heterologous host for complex fungal polyketide biosynthesis due to the availability of acetyl- and malonyl-CoA building blocks. In our pursuit to use this reservoir of polyketide precursors, we generated strains capable of producing 6-MSA and bostrycoidin while exploring possibilities of titer optimization. Successful heterologous expression of a type I PKS requires co-expression of a 4´-phosphopantetheinyltransferase (PPTase) (Wattanachaisaereekul et al., 2007; Rugbjerg et al., 2013) due to its activating effect on the PKS. Namely for FSR1, bioinformatic characterization of which has previously identified the domain architecture to consist of a starter acyl transferase (SAT), a ketosynthase (KS), a malonyl-CoA transferase (MAT), a product template (PT) domain, two acyl-carrier protein (ACP) domains and a reduction domain for product release (Brown et al., 2012; Studt et al., 2012; Frandsen et al., 2016), the PPTase activates the two ACP domains responsible for moving the growing polyketide chain between the various domains of the PKS (Majerus et al., 1965). To ensure compatibility between the FSR1 PKS and the PPTase, we chose the PPTase from *F. solani* (*FsPPT1*), which we previously used for bostrycoidin production in *S. cerevisiae* (Pedersen et al., 2022). The same PPTase was used for the expression of 6-MSAS PKS.

For 6-MSA and bostrycoidin production in *Y. lipolytica*, we chose the CRISPR-Cas9 system developed by Schwartz et al., 2017 (Schwartz et al., 2017) for targeted genomic integration of the expression cassettes. This system uses the strong constitutive hybrid promoter UAS1B8-TEF (136), which contains eight upstream activation sequence (UAS1B) elements and a 136 bp region of the TEF promoter upstream of the ATG start site (Blazeck et al., 2011). Plasmids were sequentially transformed into *Y. lipolytica*, and plasmid integration was subsequently validated via diagnostic cPCR with two or three primers (**Supplementary Material, Supplementary Figures 4A–D**).

**Figure 2:**
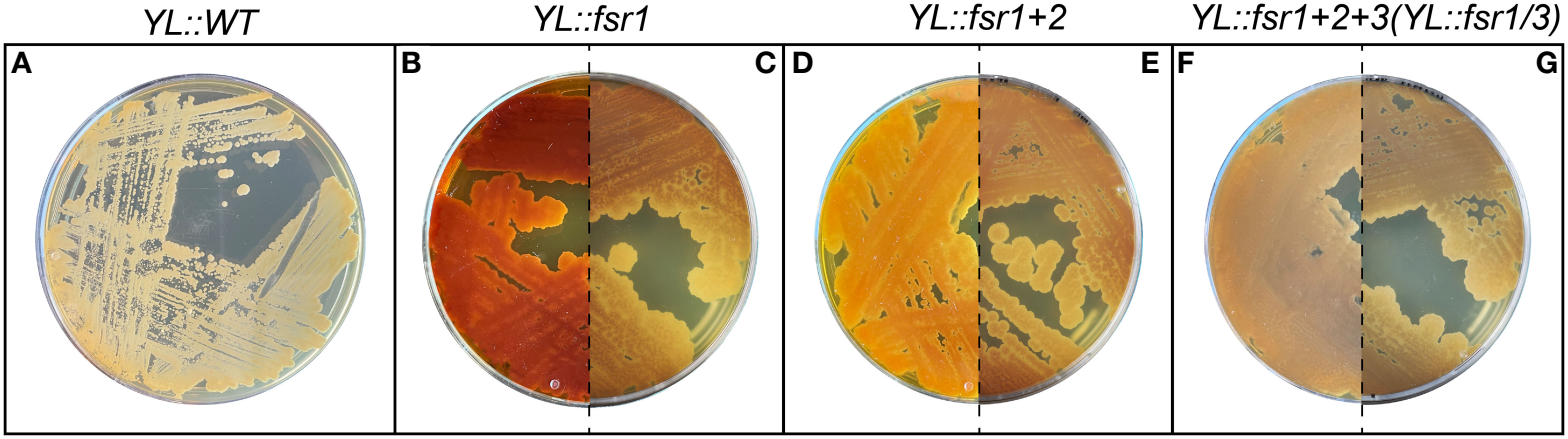
Visual confirmation of bostrycoidin biosynthetic pathway integration. **(A)** Wild-type strain of Y. lipolytica. **(B—G)** Strains expressing the bostrycoidin pathway. A difference between the non-curated **(B, D, F)** and donor-plasmid-curated strains **(C, E, G)** could be observed, confirming that Y. lipolytica requires curation with 5-FOA to eliminate the URA3-containing donor plasmid.

Due to the pigmentation of bostrycoidin and the intermediates in the pathway, a change in the color of the strains could immediately be observed after a successful transformation and genomic integration (**Figure 2**). Additionally, the *YL::fsr1/3* strain was ultimately validated via whole genome sequencing (**Supplementary Material, Supplementary Figure 5**).

We evaluated the possibility of rewiring the metabolism of *Y. lipolytica* towards preferential polyketide biosynthesis as a potential means for increasing bostrycoidin production and considered increasing acetyl- and malonyl-CoA pools by enhancing oxidation and degradation of lipids. With β-oxidation being the primary degradation process of free fatty acids (FFA) into the precursor acetyl-CoA, we targeted two key enzymes, TGL4 and AOX2, encoded by *Tg4* and *POX2*, respectively, for overexpression by inserting the UAS1B8-TEF(136) promoter in front of the two corresponding genes. We generated two CRISPR-Cas9 plasmids containing sgRNAs targeting a region of 5 bp upstream of the start codons of *Tgl4* and *POX2* and the matching donor plasmid with corresponding HR. The plasmid pairs were used to transform the *YL::fsr1/3* strain to overexpress *Tgl4* and *POX2*, alone or in combination, resulting in the strain *YL::fsr1/3+POX2, YL::fsr1/3+Tgl4*, and *YL::fsr1/3+POX2+Tgl4*, which were validated with three-primer cPCR (**Supplementary Material, Supplementary Figures 4E, F**).

### 3.2 Development of an LC-MS/MS method for 6-MSA and bostrycoidin quantification

To enable fast and reliable quantification of 6-MSA and bostrycoidin, we developed an LC-MS/MS-based, which could be directly used on the cultivation medium without tedious extraction. The parameters were obtained by automatically adjusting the selected reaction monitoring (SRM) settings (**Table 2**). 6-MSA was detected using the precursor ion ([M-H]- 150.92) and quantifier/qualifier ions (106.99/91.98), and bostrycoidin was detected using the precursor ion ([M+H]+ 286.20) and quantifier/qualifier ions (159.1/242.8).

**Table 2:** Parameters for selected reaction monitoring (SRM) transitions for 6-MSA and bostrycoidin.

	RT^a^	Precursor ion	Product ions^b^	S-lens	CE^c^
6-MSA	3.89	150.9	107.0/92.0	47	17/30
Bostrycoidin	6.20	286.1	242.9/159.1	134	28/39

^a^Retention time; ^b^Quantifier/qualifier/qualifier ions; ^c^Collision energy (V) for product ions.

**Figure 3:**
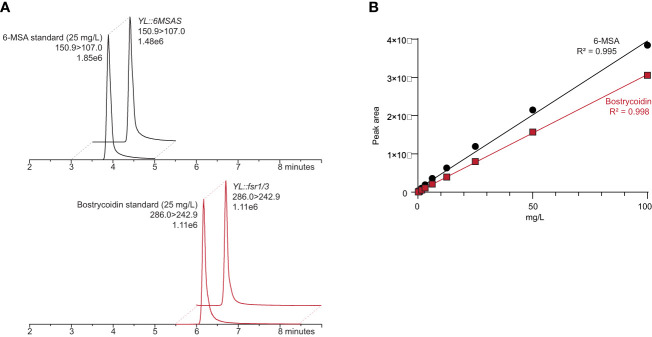
**(A)** Chromatograms of a 6-MSA and bostrycoidin standard of 25 mg/L and extracts from *Y. lipolytica* transformants, both containing 31 mg/L 6-MSA and bostrycoidin, respectively. **(B)** Standard curves of 6-MSA and bostrycoidin in a twofold dilution series (0.02 - 100 mg/L).

6-MSA and bostrycoidin were quantified as single peaks (**Figure 3A**) using the transition 150.9>107.0 and 286.1 > 242.9, respectively, in a linear manner (R^2 = ^0.995 and R^2 = ^0.998; **Figure 3B**) in the standard series (0.02 – 100 mg/L). These standard curves were subsequently used to quantify 6-MSA and bostrycoidin in the *Y. lipolytica* samples.

### 3.3 Biosynthesis of 6-MSA in *Y. lipolytica*

**Figure 4:**
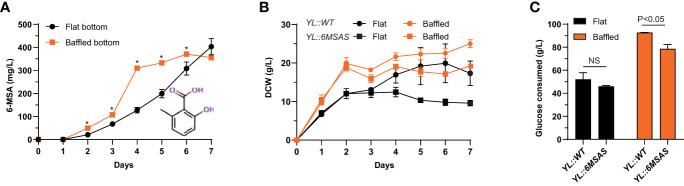
**(A)** 6-MSA production in flat vs. baffled bottom shake flasks. **(B)** Biomass comparison of wild-type and 6-MSA-producing strain, based on dry cell weight (DCW). **(C)** Comparison of glucose consumption on the last day of one-week cultivation for *YL::WT* and *YL::6MSAS* in flat and baffled bottom shake flasks. *P ≤ 0.05.

The effects of biosynthetic pathway transfers were evaluated based on product biosynthesis during shake flask fermentation in YPD (10% glucose) media. First, we investigated the production of the simple 6-MSA polyketide by transferring the PKS gene *6MSAS* from *Aspergillus hancockii* into *Y. lipolytica*. The production of 6-MSA was evaluated for biological triplicates of the *YL::6MSAS* strain grown in flat and baffled bottom shake flasks. The results showed that 6-MSA accumulated faster in the baffled flasks, and the concentration was significantly higher than in the flat bottom flasks on days 3-6. After four days, the yield was 310 mg/L and reached a maximum of 370 mg/L after six days. In the flat bottom flasks, 6-MSA was produced more slowly but amounted to 403 ± 36 mg/L after seven days (**Figure 4A**). While 6-MSA production was similar in the strains grown in either flask type, the growth patterns differed according to biomass and final glucose consumption. The wild-type strain, *YL::WT*, displayed overall a more proliferous growth, both in terms of biomass and glucose consumption measured on the 7^th^ day of cultivation. Nonetheless, for both strains, *YL::WT* and *YL::6MSAS*, growth in baffled bottom flasks led to more biomass, 25 g/L and 19.2 g/L dry cell weight (DCW), respectively, as well as a higher level of glucose consumption, with almost all glucose being consumed for *YL::WT* and 80% of the glucose consumed by the *YL::6MSAS* strain (**Figures 4B, C**).

### 3.4 Biosynthesis of bostrycoidin in *Y. lipolytica*

**Figure 5:**
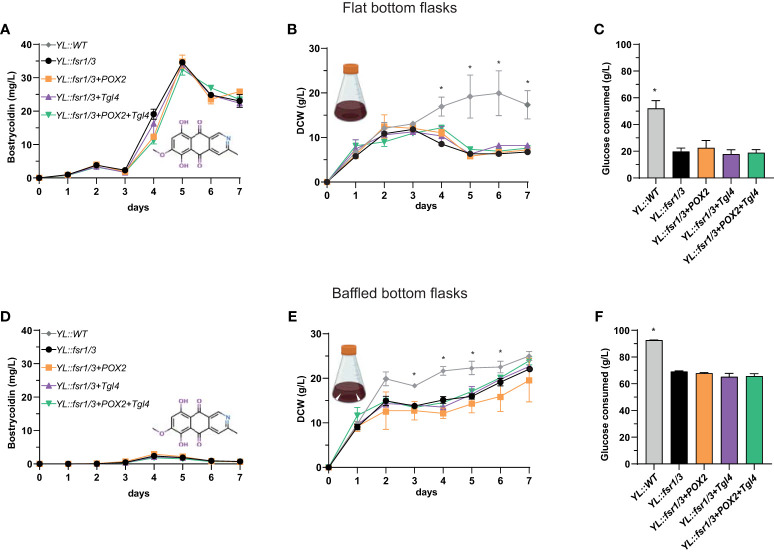
Comparison of bostrycoidin titers and strain growth in flat vs. baffled bottom shake flasks. **(A—C)** Results of one-week cultivation in flat bottom shake flasks. **(A)** Bostrycoidin titers, **(B)** DCW, **(C)** Glucose consumption; **(D, E)** Results of one-week cultivation in baffled bottom shake flasks. **(D)** Bostrycoidin titers, **(E)** DCW, **(F)** Glucose consumption. *P ≤ 0.05.

In the context of an entire biosynthetic pathway transfer, the core genes, *fsr1/fsr2/fsr3* from the fusarubin biosynthetic gene cluster (BGC) of *Fusarium solani* (PKS3 gene cluster) were transferred into *Y. lipolytica*. Additionally, the strain capable of producing bostrycoidin was modified to overexpress genes from the ß-oxidation cycle to optimize polyketide titers. Production of bostrycoidin in all the strains, including the strains with modified ß-oxidation, was evaluated based on biological triplicates grown in shake flasks with baffles or flat bottom to compare the effect on *Y. lipolytica* growth and subsequently secondary metabolite production. All strains grown in flat bottom flasks yielded significantly higher titers of bostrycoidin (**Figure 5A**). For instance, the *YL::fsr1/3* strain produced 34.6 ± 0.5 mg/L in flat bottom flasks and only 2 mg/L in the baffled flasks (**Figure 5D**). However, we did not observe an effect from manipulating β-oxidation genes, as insertion of the constitutive promoter in front of *POX2* and *Tgl4* did not significantly impact bostrycoidin yields. In parallel, the growth pattern of the bostrycoidin-producing strains was compared to the *YL::WT* strain for the two cultivation set-ups, and in both instances, the WT strain consumed more glucose and resulted in a higher DCW (**Figures 5B, C, E, F**).

## 4 Discussion

In our study, we chose bostrycoidin as the target compound for heterologous production in *Y. lipolytica* due to its promising electrochemical properties (Kristensen et al., 2020), enabling its use in a redox-flow battery to store energy from renewable sources (Wilhelmsen et al., 2023b; Wilhelmsen et al., 2023a). Simultaneously, 6-MSA is a precursor for multiple fungal secondary metabolites, including gentisyl quinone (hydroxymethyl-1, 4- benzoquinone) (Puel et al., 2010), which stood out with a relatively high positive redox potential among the other quinones with more complex structures, making it equally relevant for this study (Kristensen et al., 2020).

The 403 mg/L titers obtained for 6-MSA consolidate the position of *Y. lipolytica* as a heterologous host for non-lipid compound production, specifically polyketides. A comparison to titers of 200 mg/L (Wattanachaisaereekul et al., 2007), 367 mg/L (Hitschler and Boles, 2019), and 1.7 g/L (Kealey et al., 1998) in the heterologous host *S. cerevisiae* demonstrates a relatively similar production potential of *Y. lipolytica*. With 6-MSA as an intermediate in the biosynthetic pathways of several complex polyketide products, establishing a system that enables 6-MSA biosynthesis offers a basis for transferring the pathways into *Y. lipolytica* and producing multiple other compounds, including patulin and terreic acid (Bejenari et al., 2023). Growth monitoring through DCW and glucose measurements indicated that *YL::6MSAS* and *YL::WT* have similar growth patterns given the same cultivation conditions. While, as anticipated, the wild-type displayed a slightly more prolific growth, the results suggest that 6-MSA production does not pose a metabolic burden for the strain. 6-MSA production was not affected by the type of flasks in which the strains were cultivated. On the other hand, based on DCW and glucose consumption, better overall growth was observed in the baffled bottom flasks. The observation could be attributed to a higher level of aeration generated by the baffles since *Y. lipolytica* is a strictly aerobic organism, and increased oxygen availability improves cellular growth, leading to growth in its yeast-like form (Timoumi et al., 2017; Lopes et al., 2018).

The obtained bostrycoidin titers of approximately 35 mg/L are significantly better than the 2.2. mg/L previously obtained by our group in *S. cerevisiae* (Pedersen et al., 2020). It is, however, far from the 305 mg/L we achieved in the native host, *F. solani* (Kristensen et al., 2021), and future work will strive towards a similar output from *Y. lipolytica*. The highest bostrycoidin titers resulted from *Y. lipolytica* strains cultivated in flat bottom shake flasks, although in terms of DCW and biomass, the baffled bottom shake flasks indicated more prolific growth. We hypothesize that due to increased aeration, bostrycoidin polymerizes in the cell or the medium (Frandsen et al., 2016). In both instances, however, the higher biomass and glucose consumption levels of the wild-type strain could suggest that producing a complex polyketide, such as bostrycoidin, poses an increased burden and metabolic load on the organism. After day 5, a decrease in bostrycoidin titers was observed, which could be explained by the intracellular accumulation of bostrycoidin due to limited solubility in the media (Pedersen et al., 2020).

**Figure 6:**
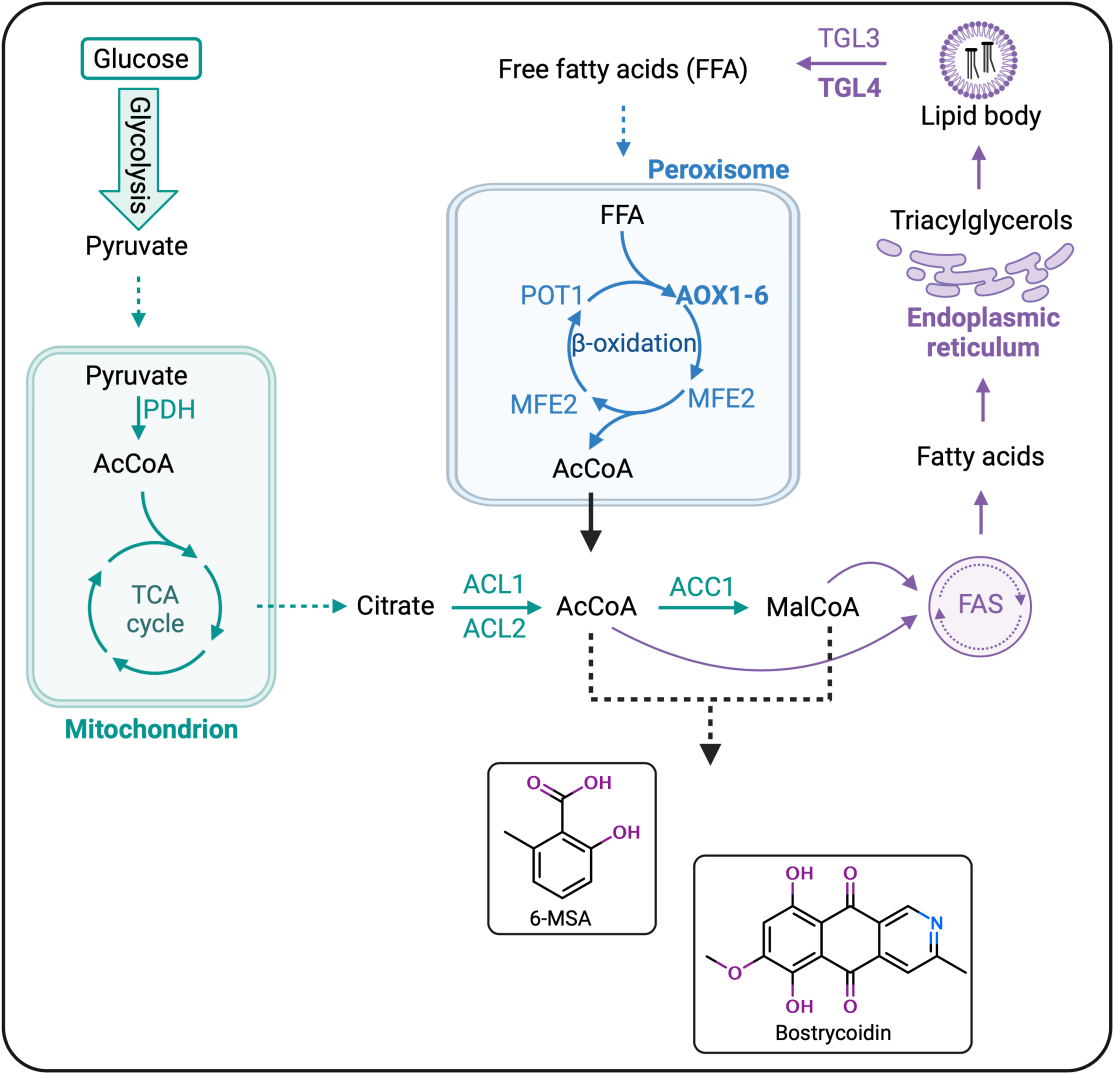
Strain engineering for optimization of 6-MSA and bostrycoidin biosynthesis in Yarrowia lipolytica. Overview of the main processes in the lipid metabolism of Y. lipolytica – the synthesis of lipid precursors, lipogenesis, and lipid degradation are color-coded green, purple, and blue, respectively. Recycling of lipid/polyketide building blocks, acetyl- and malonyl-CoA, is attempted by up-regulating ß-oxidation through overexpression of TGL4 and AOX2 (highlighted in bold). Abbreviations and enzyme names: AcCoA, acetyl-CoA; MalCoA, malonyl-CoA; PDH, pyruvate dehydrogenase complex; TCA, tricarboxylic acid; ACL1 and ACL2, ATP-citrate lyases; ACC1, acetyl-CoA carboxylase; FAS, fatty acid synthase complex; TGL3 and TGL4, lipases; AOX1-6, acyl-CoA oxidases (encoded by POX1-6 genes); MFE2, multi-functional enzyme; POT1, thiolase.

In parallel to heterologously producing these two compounds of interest in *Y. lipolytica*, we hypothesized that overexpressing key genes in the lipid β-oxidation could lead to an increased pool of acetyl- and malonyl-CoA, thereby increasing the polyketide production. For β-oxidation overexpression, we focused on bostrycoidin biosynthesis and chose *Tgl4* and *POX2* as target genes for overexpression. TGL4 is a positive regulator of the TGL3 lipase, responsible for the hydrolysis of triacylglycerols (TAGs) into free fatty acids (FFAs) that can subsequently be fed into the β-oxidation cycle, while POX2 is the most active of the six acyl-CoA oxidases (AOXs) and catalyzes the first, rate-limiting step of β-oxidation (Beopoulos et al., 2009; Dulermo et al., 2013). Therefore, by overexpressing these two genes, the expected effect was an increase in bostrycoidin titers due to an increased pool of polyketide precursors resulting from degraded FFAs (**Figure 6**).

We did not observe, however, a significant effect of the overexpression experiments on the bostrycoidin titers and hypothesize that the potential reason could lie in the transport at an efficient rate of acetyl-CoA outside the peroxisome for subsequent recycling. While the transport of acetyl-CoA between cellular compartments, i.e., export from the peroxisome and import into the mitochondria, to enter the TCA cycle after β-oxidation, is not sufficiently studied in *Y. lipolytica*, it is an essential part of the lipid metabolism that needs to be understood, to harness the full potential of this heterologous host. The aspect becomes even more relevant when considering that acetyl-CoA cannot freely traverse lipid membranes and must be transported in other forms that allow its recycling for the TCA cycle and gluconeogenesis (Liu et al., 2019a; Van Roermund et al., 1995). At least two enzymatic systems are reported in *Y. lipolytica*: the glyoxylate shunt pathway and the carnitine/acetyl-carnitine shuttle (Van Roermund et al., 1995; Fickers et al., 2005; Messina et al., 2023). The glyoxylate shunt pathway is a variation of the TCA cycle, which converts two units of acetyl-CoA into malate and succinate (C4 intermediates of the TCA cycle) (Liu et al., 2019a). Two key enzymes are involved in this process: an isocitrate lyase (encoded by *ICL1*) and a malate synthase (*MLS1* gene) (Fickers et al., 2005; Messina et al., 2023; Kujau et al., 1992). According to Messina et al., 2023 (Messina et al., 2023), the carnitine system leads to the conversion of acetyl-CoA into carnitine esters (acetylcarnitine) by a peroxisomal carnitine acetyltransferase (encoded by *pYlCat2*), which, after diffusion through the peroxisomal matrix, are transported by a mitochondrial carnitine/acetylcarnitine carrier (YlCRC1) in the mitochondria, where they are at last converted back to acetyl-CoA and free carnitine by a mitochondrial carnitine acetyltransferase (encoded by *mYLCat2*) (Van Roermund et al., 1995; Messina et al., 2023). If any of these processes happen to be a rate-limiting step, the effect could be the transport of the acetyl-CoA precursor outside the peroxisome at a too-slow rate to notice an effect on the titers of the target polyketide. Markham et al., 2018 (Markham et al., 2018) have previously used a metabolic engineering approach to redirect fluxes of acetyl- and malonyl-CoA by overexpressing the peroxisomal matrix protein Pex10, and although Pex10 is not directly involved in the β-oxidation of fatty acids, its overexpression led to an increase in TAL yield from 2.1 g/L to 2.4 g/L. Still, the most significant factor in titer optimization appeared to be the cultivation conditions, demonstrated by an increase in yield to 36 g/L upon switch to a controlled bioreactor.

With the titers of 403 mg/L for the structurally simpler 6-MSA and 35 mg/L for the complex bostrycoidin, these compounds are the first fungal polyketides, to date, produced in the heterologous host *Y. lipolytica*. In light of these results, the present study demonstrates and further supports the premise of *Y. lipolytica* being a production workhorse for both simple and complex fungal polyketides.

## Data availability statement

The original contributions presented in the study are publicly available. This data can be found here: NCBI GenBank, accession PRJNA1085342–PRJNA1085342.

## Author contributions

MB: Conceptualization, Data curation, Formal analysis, Investigation, Methodology, Software, Validation, Visualization, Writing – original draft, Writing – review & editing. ES: Conceptualization, Investigation, Writing – review & editing, Data curation, Methodology. JM: Data curation, Investigation, Writing – review & editing. AJ: Data curation, Investigation, Writing – review & editing. LC: Data curation, Investigation, Writing – review & editing. AT: Data curation, Investigation, Writing – review & editing. CA: Data curation, Investigation, Writing – review & editing. VS: Data curation, Investigation, Writing – review & editing. TP: Investigation, Writing – review & editing. MN: Investigation, Writing – review & editing. JS: Conceptualization, Data curation, Funding acquisition, Investigation, Visualization, Writing – original draft, Writing – review & editing, Supervision.

## References

[B1] Alberti F. Foster G. D. Bailey A. M. (2017). Natural products from filamentous fungi and production by heterologous expression. Appl. Microbiol. Biotechnol. 101, 493–500. doi: 10.1007/s00253-016-8034-2. 27966047 PMC5219032

[B2] André A. Chatzifragkou A. Diamantopoulou P. Sarris D. Philippoussis A. Galiotou-Panayotou M.. (2009). Biotechnological conversions of bio-diesel-derived crude glycerol by Yarrowia lipolytica strains. Eng. Life Sci. 9, 468–478. doi: 10.1002/elsc.200900063

[B3] Barth G. Gaillardin C. (1997). Physiology and genetics of the dimorphic fungus Yarrowia lipolytica. FEMS Microbiol. Rev. 19, 219–237. doi: 10.1111/j.1574-6976.1997.tb00299.x. 9167256

[B4] Bedford D. J. Schweizer E. Hopwood D. A. Khosla C. (1995). Expression of a functional fungal polyketide synthase in the bacterium Streptomyces coelicolor A3(2). J. Bacteriol 177, 4544–4548. doi: 10.1128/jb.177.15.4544-4548.1995. 7635840 PMC177212

[B5] Bejenari M. Sondergaard T. E. Sørensen J. L. (2023). 6-MSA, a secondary metabolite distribution hub with multiple fungal destinations. J. Appl. Microbiol. 134, 1–9. doi: 10.1093/jambio/lxad107. 37218693

[B6] Beopoulos A. Cescut J. Haddouche R. Uribelarrea J. L. Molina-Jouve C. Nicaud J. M. (2009). Yarrowia lipolytica as a model for bio-oil production. Prog. Lipid Res. 48, 375–387. doi: 10.1016/j.plipres.2009.08.005. 19720081

[B7] Blazeck J. Liu L. Redden H. Alper H. (2011). Tuning gene expression in yarrowia lipolytica by a hybrid promoter approach. Appl. Environ. Microbiol. 77, 7905–7914. doi: 10.1128/AEM.05763-11. 21926196 PMC3208987

[B8] Blazeck J. Reed B. Garg R. Gerstner R. Pan A. Agarwala V.. (2013). Generalizing a hybrid synthetic promoter approach in Yarrowia lipolytica. Appl. Microbiol. Biotechnol. 97, 3037–3052. doi: 10.1007/s00253-012-4421-5. 23053080

[B9] Bond C. Tang Y. Li L. (2016). Saccharomyces cerevisiae as a tool for mining, studying and engineering fungal polyketide synthases. Fungal Genet. Biol. 89, 52–61. doi: 10.1016/j.fgb.2016.01.005. 26850128 PMC4789138

[B10] Bredeweg E. L. Pomraning K. R. Dai Z. Nielsen J. Kerkhoven E. J. Baker S. E. (2017). A molecular genetic toolbox for Yarrowia lipolytica. Biotechnol. Biofuels 10, 2. doi: 10.1186/s13068-016-0687-7. 28066508 PMC5210315

[B11] Brown D. W. Butchko R. A. E. Busman M. Proctor R. H. (2012). Identification of gene clusters associated with fusaric acid, fusarin, and perithecial pigment production in Fusarium verticillioides. Fungal Genet. Biol. 49, 521–532. doi: 10.1016/j.fgb.2012.05.010. 22652150

[B12] Brown D. W. Proctor R. H. (2016). Insights into natural products biosynthesis from analysis of 490 polyketide synthases from Fusarium. Fungal Genet. Biol. 89, 37–51. doi: 10.1016/j.fgb.2016.01.008. 26826610

[B13] Cardenas J. Da Silva N. A. (2016). Engineering cofactor and transport mechanisms in Saccharomyces cerevisiae for enhanced acetyl-CoA and polyketide biosynthesis. Metab. Eng. 36, 80–89. doi: 10.1016/j.ymben.2016.02.009. 26969250

[B14] Celińska E. Ledesma-Amaro R. Larroude M. Rossignol T. Pauthenier C. Nicaud J. M. (2017). Golden Gate Assembly system dedicated to complex pathway manipulation in Yarrowia lipolytica. Microb. Biotechnol. 10, 450–455. doi: 10.1111/1751-7915.12605. 28217858 PMC5328822

[B15] Chen Y. Daviet L. Schalk M. Siewers V. Nielsen J. (2013). Establishing a platform cell factory through engineering of yeast acetyl-CoA metabolism. Metab. Eng. 15, 48–54. doi: 10.1016/j.ymben.2012.11.002. 23164578

[B16] Curran K. A. Morse N. J. Markham K. A. Wagman A. M. Gupta A. Alper H. S. (2015). Short synthetic terminators for improved heterologous gene expression in yeast. ACS Synth Biol. 4, 824–832. doi: 10.1021/sb5003357. 25686303

[B17] Doench J. G. Fusi N. Sullender M. Hegde M. Vaimberg E. W. Donovan K. F.. (2016). Optimized sgRNA design to maximize activity and minimize off-target effects of CRISPR-Cas9. Nat. Biotechnol. 34, 184–191. doi: 10.1038/nbt.3437. 26780180 PMC4744125

[B18] Dulermo T. Tréton B. Beopoulos A. Gnankon A. P. K. Haddouche R. Nicaud J. M. (2013). Characterization of the two intracellular lipases of Y. lipolytica encoded by TGL3 and TGL4 genes: New insights into the role of intracellular lipases and lipid body organisation. Biochim. Biophys. Acta - Mol. Cell Biol. Lipids 1831, 1486–1495. doi: 10.1016/j.bbalip.2013.07.001. 23856343

[B19] Fickers P. Benetti P. H. Waché Y. Marty A. Mauersberger S. Smit M. S.. (2005). Hydrophobic substrate utilisation by the yeast Yarrowia lipolytica, and its potential applications. FEMS Yeast Res. 5, 527–543. doi: 10.1016/j.femsyr.2004.09.004. 15780653

[B20] Frandsen R. J. N. Rasmussen S. A. Knudsen P. B. Uhlig S. Petersen D. Lysøe E.. (2016). Black perithecial pigmentation in Fusarium species is due to the accumulation of 5-deoxybostrycoidin-based melanin. Sci. Rep. 6, 26206. doi: 10.1038/srep26206. 27193384 PMC4872168

[B21] Harvey C. J. B. Tang M. Schlecht U. Horecka J. Fischer C. R. Lin H. C.. (2018). HEx: A heterologous expression platform for the discovery of fungal natural products. Sci. Adv. 4, eaar5459. doi: 10.1126/sciadv.aar5459 29651464 PMC5895447

[B22] Hertweck C. (2009). The biosynthetic logic of polyketide diversity. Angew Chemie - Int. Ed 48, 4688–4716. doi: 10.1002/anie.200806121. 19514004

[B23] Hertweck C. Luzhetskyy A. Rebets Y. Bechthold A. (2007). Type II polyketide synthases: Gaining a deeper insight into enzymatic teamwork. Nat. Prod Rep. 24, 162–190. doi: 10.1039/B507395M. 17268612

[B24] Hitschler J. Boles E. (2019). *De novo* production of aromatic m-cresol in Saccharomyces cerevisiae mediated by heterologous polyketide synthases combined with a 6-methylsalicylic acid decarboxylase. Metab. Eng. Commun. 9, e00093. doi: 10.1016/j.mec.2019.e00093. 31193192 PMC6520567

[B25] Holkenbrink C. Dam M. I. Kildegaard K. R. Beder J. Dahlin J. Doménech Belda D.. (2018). EasyCloneYALI: CRISPR/cas9-based synthetic toolbox for engineering of the yeast yarrowia lipolytica. Biotechnol. J. 13, 1700543. doi: 10.1002/biot.201700543. 29377615

[B26] Hsu P. D. Scott D. A. Weinstein J. A. Ran F. A. Konermann S. Agarwala V.. (2013). DNA targeting specificity of RNA-guided Cas9 nucleases. Nat. Biotechnol. 31, 827–832. doi: 10.1038/nbt.2647. 23873081 PMC3969858

[B27] Kealey J. T. Liu L. Santi D. V. Betlach M. C. Barr P. J. (1998). Production of a polyketide natural product in nonpolyketide-producing prokaryotic and eukaryotic hosts. Proc. Natl. Acad. Sci. 95, 505–509. doi: 10.1073/pnas.95.2.505. 9435221 PMC18449

[B28] Kjærbølling I. Mortensen U. H. Vesth T. Andersen M. R. (2019). Strategies to establish the link between biosynthetic gene clusters and secondary metabolites. Fungal Genet. Biol. 130, 107–121. doi: 10.1016/j.fgb.2019.06.001. 31195124

[B29] Kristensen S. B. Pedersen T. B. Nielsen M. R. Wimmer R. Muff J. Sørensen J. L. (2021). Production and selectivity of key fusarubins from fusarium solani due to media composition. Toxins (Basel) 13, 1–12. doi: 10.3390/toxins13060376. PMC8230112 34070644

[B30] Kristensen S. B. van Mourik T. Pedersen T. B. Sørensen J. L. Muff J. (2020). Simulation of electrochemical properties of naturally occurring quinones. Sci. Rep. 10, 13571. doi: 10.1038/s41598-020-70522-z. 32782387 PMC7419317

[B31] Kujau M. Weber H. Barth G. (1992). Characterization of mutants of the yeast Yarrowia lipolytica defective in acetyl-coenzyme A synthetase. Yeast 8, 193–203. doi: 10.1002/yea.320080305. 1349449

[B32] Lian J. Si T. Nair N. U. Zhao H. (2014). Design and construction of acetyl-CoA overproducing Saccharomyces cerevisiae strains. Metab. Eng. 24, 139–149. doi: 10.1016/j.ymben.2014.05.010. 24853351

[B33] Liu H. Marsafari M. Deng L. Xu P. (2019a). Understanding lipogenesis by dynamically profiling transcriptional activity of lipogenic promoters in Yarrowia lipolytica. Appl. Microbiol. Biotechnol. 103, 3167–3179. doi: 10.1007/s00253-019-09664-8. 30734122

[B34] Liu H. Marsafari M. Wang F. Deng L. Xu P. (2019b). Engineering acetyl-CoA metabolic shortcut for eco-friendly production of polyketides triacetic acid lactone in Yarrowia lipolytica. Metab. Eng. 56, 60–68. doi: 10.1016/j.ymben.2019.08.017. 31470116

[B35] Lopes M. Gomes A. S. Silva C. M. Belo I. (2018). Microbial lipids and added value metabolites production by Yarrowia lipolytica from pork lard. J. Biotechnol. 265, 76–85. doi: 10.1016/j.jbiotec.2017.11.007. 29141191

[B36] Lv Y. Marsafari M. Koffas M. Zhou J. Xu P. (2019). Optimizing oleaginous yeast cell factories for flavonoids and hydroxylated flavonoids biosynthesis. ACS Synth Biol. 8, 2514–2523. doi: 10.1021/acssynbio.9b00193. 31622552

[B37] Ma J. Gu Y. Marsafari M. Xu P. (2020). Synthetic biology, systems biology, and metabolic engineering of Yarrowia lipolytica toward a sustainable biorefinery platform. J. Ind. Microbiol. Biotechnol. 47, 845–862. doi: 10.1007/s10295-020-02290-8. 32623653

[B38] Madzak C. Blanchin-Roland S. Cordero Otero R. R. Gaillardin C. (1999). Functional analysis of upstream regulating regions from the Yarrowia lipolytica XPR2 promoter. Microbiology 145, 75–87. doi: 10.1099/13500872-145-1-75. 10206713

[B39] Madzak C. Tréton B. Blanchin-Roland S. (2000). Strong hybrid promoters and integrative expression/secretion vectors for quasi-constitutive expression of heterologous proteins in the yeast Yarrowia lipolytica. J. Mol. Microbiol. Biotechnol. 2, 207–216. 10939246

[B40] Majerus P. W. Alberts A. W. Vagelos P. R. (1965). Acyl Carrier Protein, IV. The Identification of 4´-Phosphopantetheine as the prosthetic group of the Acyl Carrier Protein. Proc. Natl. Acad. Sci. U.S.A. 53, 410–417. doi: 10.1073/pnas.53.2.410. 14294075 PMC219528

[B41] Markham K. A. Alper H. S. (2018). Synthetic biology expands the industrial potential of yarrowia lipolytica. Trends Biotechnol. 36, 1085–1095. doi: 10.1016/j.tibtech.2018.05.004. 29880228

[B42] Markham K. A. Palmer C. M. Chwatko M. Wagner J. M. Murray C. Vazquez S.. (2018). Rewiring Yarrowia lipolytica toward triacetic acid lactone for materials generation. Proc. Natl. Acad. Sci. 115, 2096–2101. doi: 10.1073/pnas.1721203115. 29440400 PMC5834725

[B43] McDaniel R. Ebert-Khosla S. Hopwood D. A. Khosla C. (1994). Engineered Biosynthesis of Novel Polyketides: actVII and actIV Genes Encode Aromatase and Cyclase Enzymes, Respectively. J. Am. Chem. Soc. 116, 10855–10859. doi: 10.1021/ja00103a001.

[B44] Messina E. de Souza C. P. Cappella C. Barile S. N. Scarcia P. Pisano I.. (2023). Genetic inactivation of the Carnitine/Acetyl-Carnitine mitochondrial carrier of Yarrowia lipolytica leads to enhanced odd-chain fatty acid production. Microb. Cell Fact 22, 128. doi: 10.1186/s12934-023-02137-8. 37443049 PMC10339547

[B45] Miller K. K. Alper H. S. (2019). Yarrowia lipolytica: more than an oleaginous workhorse. Appl. Microbiol. Biotechnol. 103, 9251–9262. doi: 10.1007/s00253-019-10200-x. 31686142

[B46] Muhammad A. Feng X. Rasool A. Sun W. Li C. (2020). Production of plant natural products through engineered Yarrowia lipolytica. Biotechnol. Adv. 43, 107555. doi: 10.1016/j.biotechadv.2020.107555. 32422161

[B47] Nielsen M. R. Sondergaard T. E. Giese H. Sørensen J. L. (2019). Advances in linking polyketides and non-ribosomal peptides to their biosynthetic gene clusters in Fusarium. Curr. Genet. 65, 1263–1280. doi: 10.1007/s00294-019-00998-4. 31139896

[B48] Palmer C. M. Miller K. K. Nguyen A. Alper H. S. (2020). Engineering 4-coumaroyl-CoA derived polyketide production in Yarrowia lipolytica through a β-oxidation mediated strategy. Metab. Eng. 57, 174–181. doi: 10.1016/j.ymben.2019.11.006. 31740389

[B49] Papanikolaou S. Aggelis G. (2002). Lipid production by Yarrowia lipolytica growing on industrial glycerol in a single-stage continuous culture. Bioresour Technol. 82, 43–49. doi: 10.1016/S0960-8524(01)00149-3. 11848376

[B50] Pedersen T. B. Nielsen M. R. Kristensen S. B. Spedtsberg E. M. L. Sørensen T. Petersen C.. (2022). Speed dating for enzymes! Finding the perfect phosphopantetheinyl transferase partner for your polyketide synthase. Microb. Cell Fact 21, 1–10. doi: 10.1186/s12934-021-01734-9. 35012550 PMC8751348

[B51] Pedersen T. B. Nielsen M. R. Kristensen S. B. Spedtsberg E. M. L. Yasmine W. Matthiesen R.. (2020). Heterologous expression of the core genes in the complex fusarubin gene cluster of Fusarium Solani. Int. J. Mol. Sci. 21, 1–10. doi: 10.3390/ijms21207601. PMC7589453 33066643

[B52] Petersen C. Sørensen T. Westphal K. R. Fechete L. I. Sondergaard T. E. Sørensen J. L.. (2022). High molecular weight DNA extraction methods lead to high quality filamentous ascomycete fungal genome assemblies using Oxford Nanopore sequencing. Microb. Genomics 8, 1–13. doi: 10.1099/mgen.0.000816. PMC9453082 35438621

[B53] Puel O. Galtier P. Oswald I. P. (2010). Biosynthesis and toxicological effects of patulin. Toxins (Basel) 2, 613–631. doi: 10.3390/toxins2040613. 22069602 PMC3153204

[B54] Raab D. Graf M. Notka F. Schödl T. Wagner R. (2010). The GeneOptimizer Algorithm: Using a sliding window approach to cope with the vast sequence space in multiparameter DNA sequence optimization. Syst. Synth Biol. 4, 215–225. doi: 10.1007/s11693-010-9062-3. 21189842 PMC2955205

[B55] Rugbjerg P. Naesby M. Mortensen U. H. Frandsen R. J. N. (2013). Reconstruction of the biosynthetic pathway for the core fungal polyketide scaffold rubrofusarin in Saccharomyces cerevisiae. Microb. Cell Fact 12, 1–9. doi: 10.1186/1475-2859-12-31. 23557488 PMC3654996

[B56] Rushing B. R. Selim M. I. (2019). Aflatoxin B1: A review on metabolism, toxicity, occurrence in food, occupational exposure, and detoxification methods. Food Chem. Toxicol. 124, 81–100. doi: 10.1016/j.fct.2018.11.047. 30468841

[B57] Sáez-Sáez J. Wang G. Marella E. R. Sudarsan S. Cernuda Pastor M. Borodina I. (2020). Engineering the oleaginous yeast Yarrowia lipolytica for high-level resveratrol production. Metab. Eng. 62, 51–61. doi: 10.1016/j.ymben.2020.08.009. 32818629 PMC7672257

[B58] Schwartz C. Cheng J. F. Evans R. Wagner J. Anglin S. Beitz A.. (2019). Validating genome-wide CRISPR-Cas9 function improves screening in the oleaginous yeast Yarrowia lipolytica. Metab. Eng. 55, 102–110. doi: 10.1016/j.ymben.2019.06.007. 31216436

[B59] Schwartz C. Hussain M. S. Blenner M. Wheeldon I. (2016). Synthetic RNA polymerase III promoters facilitate high-efficiency CRISPR-cas9-mediated genome editing in yarrowia lipolytica. ACS Synth Biol. 5, 356–359. doi: 10.1021/acssynbio.5b00162. 26714206

[B60] Schwartz C. Shabbir-Hussain M. Frogue K. Blenner M. Wheeldon I. (2017). Standardized markerless gene integration for pathway engineering in yarrowia lipolytica. ACS Synth Biol. 6, 402–409. doi: 10.1021/acssynbio.6b00285. 27989123

[B61] Schwartz C. Wheeldon I. (2018). “CRISPR-cas9-mediated genome editing and transcriptional control in yarrowia lipolytica,” in Synthetic Biology: Methods and Protocols. Ed. Braman J. C. (Springer New York, New York, NY), 327–345. 10.1007/978-1-4939-7795-6_18 29754237

[B62] Shabbir Hussain M. Gambill L. Smith S. Blenner M. A. (2016). Engineering promoter architecture in oleaginous yeast yarrowia lipolytica. ACS Synth Biol. 5, 213–223. doi: 10.1021/acssynbio.5b00100. 26635071

[B63] Shimizu Y. Ogata H. Goto S. (2017). Type III polyketide synthases: Functional classification and phylogenomics. ChemBioChem 18, 50–65. doi: 10.1002/cbic.201600522. 27862822

[B64] Staunton J. Weissman K. J. (2001). Polyketide biosynthesis: A millennium review. Nat. Prod Rep. 18, 380–416. doi: 10.1039/a909079g. 11548049

[B65] Studt L. Wiemann P. Kleigrewe K. Humpf H. U. Tudzynski B. (2012). Biosynthesis of fusarubins accounts for pigmentation of Fusarium fujikuroi perithecia. Appl. Environ. Microbiol. 78, 4468–4480. doi: 10.1128/AEM.00823-12. 22492438 PMC3370568

[B66] Timoumi A. Bideaux C. Guillouet S. E. Allouche Y. Molina-Jouve C. Fillaudeau L.. (2017). Influence of oxygen availability on the metabolism and morphology of Yarrowia lipolytica: insights into the impact of glucose levels on dimorphism. Appl. Microbiol. Biotechnol. 101, 7317–7333. doi: 10.1007/s00253-017-8446-7. 28879478

[B67] Tippelt A. Nett M. (2021). Saccharomyces cerevisiae as host for the recombinant production of polyketides and nonribosomal peptides. Microb. Cell Fact 20, 1–24. doi: 10.1186/s12934-021-01650-y. 34412657 PMC8374128

[B68] Tobert J. A. (2003). Lovastatin and beyond: The history of the HMG-CoA reductase inhibitors. Nat. Rev. Drug Discovery 2, 517–526. doi: 10.1038/nrd1112. 12815379

[B69] Untergasser A. Cutcutache I. Koressaar T. Ye J. Faircloth B. C. Remm M.. (2012). Primer3—new capabilities and interfaces. Nucleic Acids Res. 40, e115–e115. doi: 10.1093/nar/gks596. 22730293 PMC3424584

[B70] Van Roermund C. W. T. Elgersma Y. Singh N. Wanders R. J. A. Tabak H. F. (1995). The membrane of peroxisomes in Saccharomyces cerevisiae is impermeable to NAD(H) and acetyl-CoA under in vivo conditions. EMBO J. 14, 3480–3486. doi: 10.1002/embj.1995.14.issue-14. 7628449 PMC394415

[B71] Wattanachaisaereekul S. Lantz A. E. Nielsen M. L. Andrésson Ó.S. Nielsen J. (2007). Optimization of heterologous production of the polyketide 6-MSA in Saccharomyces cerevisiae. Biotechnol. Bioeng 97, 893–900. doi: 10.1002/bit.21286. 17171715

[B72] Wattanachaisaereekul S. Lantz A. E. Nielsen M. L. Nielsen J. (2008). Production of the polyketide 6-MSA in yeast engineered for increased malonyl-CoA supply. Metab. Eng. 10, 246–254. doi: 10.1016/j.ymben.2008.04.005. 18555717

[B73] Wilhelmsen C. O. Kristensen S. B. Nolte O. Volodin I. A. Christiansen J. V. Isbrandt T.. (2023a). Demonstrating the use of a fungal synthesized quinone in a redox flow battery. Batter Supercaps 6, e202200365. doi: 10.1002/batt.202200365.

[B74] Wilhelmsen C. O. Muff J. Sørensen J. L. (2023b). Are biologically synthesized electrolytes the future in green energy storage? Energy Storage 5(6), e450. doi: 10.1002/est2.450.

[B75] Wong L. Engel J. Jin E. Holdridge B. Xu P. (2017). YaliBricks, a versatile genetic toolkit for streamlined and rapid pathway engineering in Yarrowia lipolytica. Metab. Eng. Commun. 5, 68–77. doi: 10.1016/j.meteno.2017.09.001. 29188186 PMC5699529

[B76] Xiong X. Chen S. (2020). Expanding toolbox for genes expression of yarrowia lipolytica to include novel inducible, repressible, and hybrid promoters. ACS Synth Biol. 9, 2208–2213. doi: 10.1021/acssynbio.0c00243. 32584553

[B77] Yu J. Landberg J. Shavarebi F. Bilanchone V. Okerlund A. Wanninayake U.. (2018). Bioengineering triacetic acid lactone production in Yarrowia lipolytica for pogostone synthesis. Biotechnol. Bioeng 115, 2383–2388. doi: 10.1002/bit.26733. 29777591 PMC6855914

