# Supplementary material for: First-class – biosynthesis of 6-MSA and bostrycoidin type I polyketides in Yarrowia lipolytica
Source: Front Fungal Biol. 2024 Mar 22;5:1327777. doi: 10.3389/ffunb.2024.1327777 (PMC10995274; doi:10.3389/ffunb.2024.1327777)
Supplement: Supplementary file 1 [file DataSheet_1.pdf]

Supplementary material

Supplementary Table 1. Primer list for biosynthetic pathway transfer

Name	Sequence	Purpose
	**Bostrycoidin**	
G020	CCTCATGTAATTAGTTATGTCACGCT	Amplification of donor plasmid backbones
G021	CCTTTGAATGATTCTTATACTCAGAAGGA	
H057	**TTCGGAGAGGACGCCACCGACGCTCTGTAA**CCTCATGTAATTAGTTATGTCACGCT	Amplification of pD17 for *FsPPT1* insertion
H058	**CTGGATCACGGTAGGGGTAGACTCGCCCAT**CCTTTGAATGATTCTTATACTCAGAAGG A	
H053	ATGGGCGAGTCTACCCCTAC	Amplification of *FsPPT1*
H054	TTACAGAGCGTCGGTGGC	
G072	**TTCCTTCTGAGTATAAGAATCATTCAAAGG**ATGACCGACAACCTGAAGCTGTA	Amplification of *fsr1* (part I)
I037	AGATCACAGAAGGAGCGGAC	
I036	CGACCTAAGTCCGCTCCT	Amplification of *fsr1* (part II)
G073	**TGTAAGCGTGACATAACTAATTACATGAGG**TTACACTCGGGGTCCCCACA	
G074	**TTCCTTCTGAGTATAAGAATCATTCAAAGG**ATGCACAAGACCGAGCGAGAT	Amplification of *fsr2*
G075	**TGTAAGCGTGACATAACTAATTACATGAGG**AGCGTGGCCGTTCAGG	
G076	**TTCCTTCTGAGTATAAGAATCATTCAAAGG**ATGCAGATCAACGACCAGACC	Amplification of *fsr3*
G077	**TGTAAGCGTGACATAACTAATTACATGAGG**TTAGGCCCAGTCGCCGTC	
G032	ATAGCCACGACCACCAAGTC	Validation of *FsPPT1* genomic integration
G082	ATGGGCGAGTCTACCCCTAC	
G083	CTCGGTCAGCAGAGACAGG	
G035	CCAGACCCTTTGCGTACAAT	
G028	CCAAACCAACAAGGCCTCTA	Validation of *fsr1* genomic integration
G029	GGCTAAAGTCCCAACCACAA	
G081	ACGAGCTGGTGCAGAAGTAC	
G031	CACGACCCAGACCGTATCTT	
I068	GCAGTCTTGGCCGTCTTAAC	Validation of *fsr2* genomic integration
I069	AGGAGGTGCTCGACGTTAGA	
I070	TTGTGGTTGGGACTTTAGCC	
I071	GTCAAGACAAGCCTGCAACA	
G084	GCCACATTAGAGACCCCAAG	Validation of *fsr3* genomic integration
G085	TTCCTCGCTTGATAGAGTCCTC	
G086	GCAAGAGCAGATCCAGGCT	
G087	AAGAGCTGTGTTTTGGAAGGTA	
J041	CTGCAGCCTAGAAGCTTTTGT	General primers for Sanger sequencing of assembled donor plasmids
J042	CGTCCCAAAACCTTCTCAAG	
	**6-MSA**	
K067	**TTCCTTCTGAGTATAAGAATCATTCAAAGG**ATGGGAGACTACAAGTCTTCCC	Amplification of *6MSAS*
K068	**TGTAAGCGTGACATAACTAATTACATGAGG**CTACTTAGCCTGGCCCTCG	
I092	CTCGAAGGCTTTAATTTGCCCTA	General forward primer for integration validation via three-primer cPCR
J041	CTGCAGCCTAGAAGCTTTTGT	General primers for Sanger sequencing of assembled donor plasmids
J042	CGTCCCAAAACCTTCTCAAG	

* Primer tails are marked in bold

Supplementary Table 2. Primer list for overexpression of β-oxidation

Name	Sequence	Purpose
	**Donor plasmid assembly and validation**	
J026	**ATCATGGTCATAGATCTAGCTGTTTCCTG**TGTGAAATTGTTATCCGCTCA	Addition of new restriction site to the pAXP plasmid
J027	**TACAACCACTTTTCCTAGGGCAAATT**CGCGCCTTTGAATGATTCTT	
J044	**CTAGATCTATGACCATGATTACGCCAAGCT**TAATTGCATCCAACAATGTTGAACCT	Removal of *hrGFP* and CYC1
J028	**AATTTGCCCTAGGAAAAGTGGTTGTA**CCAGAAAACAGATCCACAAGTACC	
J093	**CACGACAATTCTGTATCTCTTTATGTTAAC**ATAATCATCGCAATGGCACA	Amplification of *Tgl4* HR1
J091	**CCTCAGGAATTCGGGTACCACTAGT**GGCAGGGGTAAATGAGACTG	
J094	**AATCATTCAAAGGCGCGAATTTGCCCTAGG**AGAGTTTCCGAAGCAAGCAC	Amplification of *Tgl4* HR2
J096	**AACAATTTCACACAGGAAACAGCTAGATCT**TGTTGTCGAGTAGCGATCCA	
J099	**CACGACAATTCTGTATCTCTTTATGTTAAC**CCCAATCACATGCTCAGAAA	Amplification of *POX2* HR1
J097	**CCTCAGGAATTCGGGTACCACTAGT**GGTTAAGGTTGCCCGTGTAG	
J100	**AATCATTCAAAGGCGCGAATTTGCCCTAGG**CCATGAACCCCAACAACAC	Amplification of *POX2* HR2
K002	**AACAATTTCACACAGGAAACAGCTAGATCT**ATGGTGATGAATCGCTTTCC	
J046	GCCTCTTCGCTATTACGCCA	Validation of *Tgl4* HR1 insertion in the plasmid
K003	GTATTGCCCCGTTTCTACGA	
K004	CACAGCCTCAAGGTCGGTAT	Validation of *Tgl4* HR2 insertion in the plasmid
J049	TTGGAGCGAACGACCTACAC	
K005	GATAGATGGAGTGCGGAGGA	Validation of *POX2* HR1 insertion in the plasmid, combined with J46
K006	GAGGATGTCTCGGTCTGCTC	Validation of *POX2* HR2 insertion in the plasmid, combined with J49
J041	CTGCAGCCTAGAAGCTTTTGT	General primers for Sanger sequencing of assembled donor plasmids
J042	CGTCCCAAAACCTTCTCAAG	
	**sgRNA oligonucleotides**	
J055	GGGTCGGCGCAGGTTGACGTTGCATGTACACACTGTAGGGGTTTTAGAGCTAGAAATAGC	*Tgl4* sgRNA oligonucleotides, on-target score=66
J056	GCTATTTCTAGCTCTAAAACCCCTACAGTGTGTACATGCAACGTCAACCTGCGCCGACCC	
J067	GGGTCGGCGCAGGTTGACGTCGTTGCTTGTGTGATTTTTGGTTTTAGAGCTAGAAATAGC	*POX2* sgRNA oligonucleotides, on-target score=41
J068	GCTATTTCTAGCTCTAAAACCAAAAATCACACAAGCAACGACGTCAACCTGCGCCGACCC	
	**Validation of CRISPRyl plasmid and genomic integration**	
J054	TCTTCTCCACATTTTATGCTCAGTG	General reverse primer used for CRISPRyl validation via Sanger sequencing
J041	CTGCAGCCTAGAAGCTTTTGT	General forward primer for integration validation via three-primer cPCR
K099	TTGAAACCGAGCCATTGAAG	Validation of UAS1B8-TEF promoter genomic integration upstream *Tgl4*
K100	AGATGCCAGCAGACGATACC	
K055	CTCACGTGACCCGTCTCC	Validation of UAS1B8-TEF promoter genomic integration upstream *POX2*
K056	GGTGGTGGAGAACTGTCGTC	

* Primer tails are marked in bold, and sgRNA sequences are marked in red

Supplementary Table 3. sgRNA sequences corresponding to the A08, D17, AXP, and XPR2 loci (Schwartz et al., 2017)

**Locus**	**sgRNA**
**A08**	GAGTACGGCATTGATTCAGA
**D17**	TCCGTAATATAGGTGACGAC
**AXP**	GACCAGGTCGAAGTAGCTGG
**XPR2**	GCTGGACTCTCTGGTCGACG

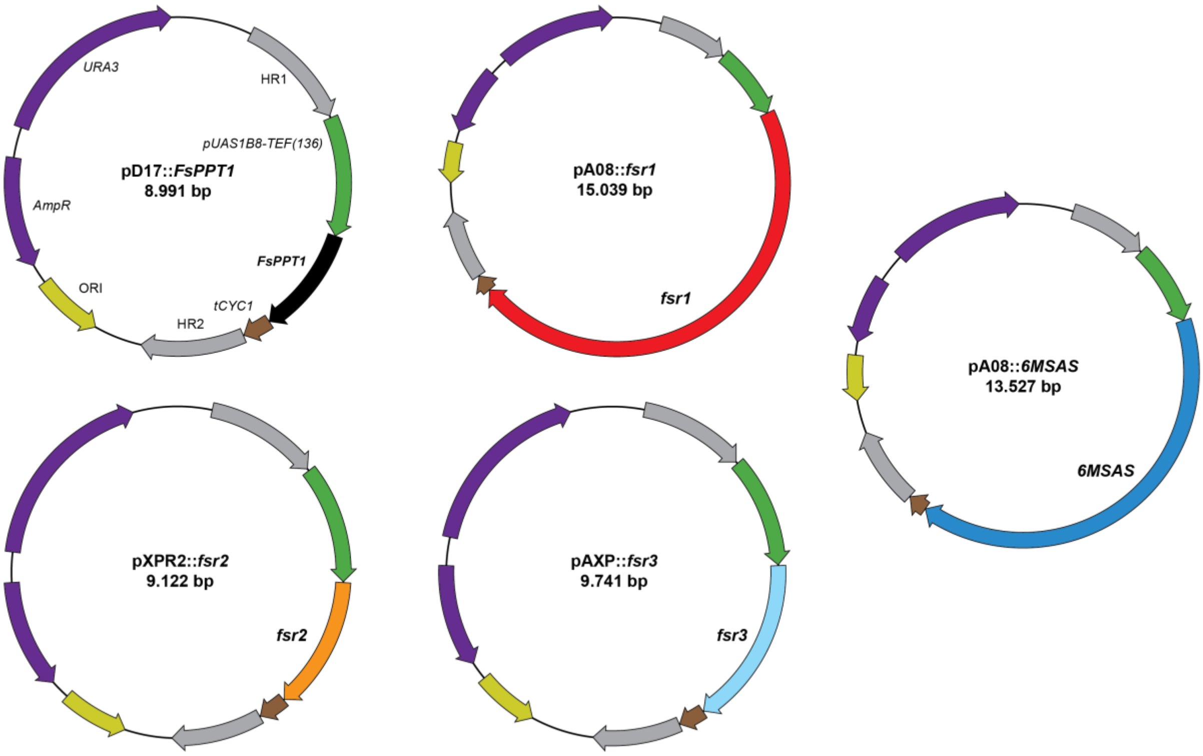

Supplemetary Figure 1. Maps of the five donor plasmids generated in this study to integrate *FsPPT1*, *fsr1/6MSAS*, *fsr2*, and *fsr3* into D17, A08, XPR2, and AXP loci, respectively, of *Yarrowia lipolytica*.

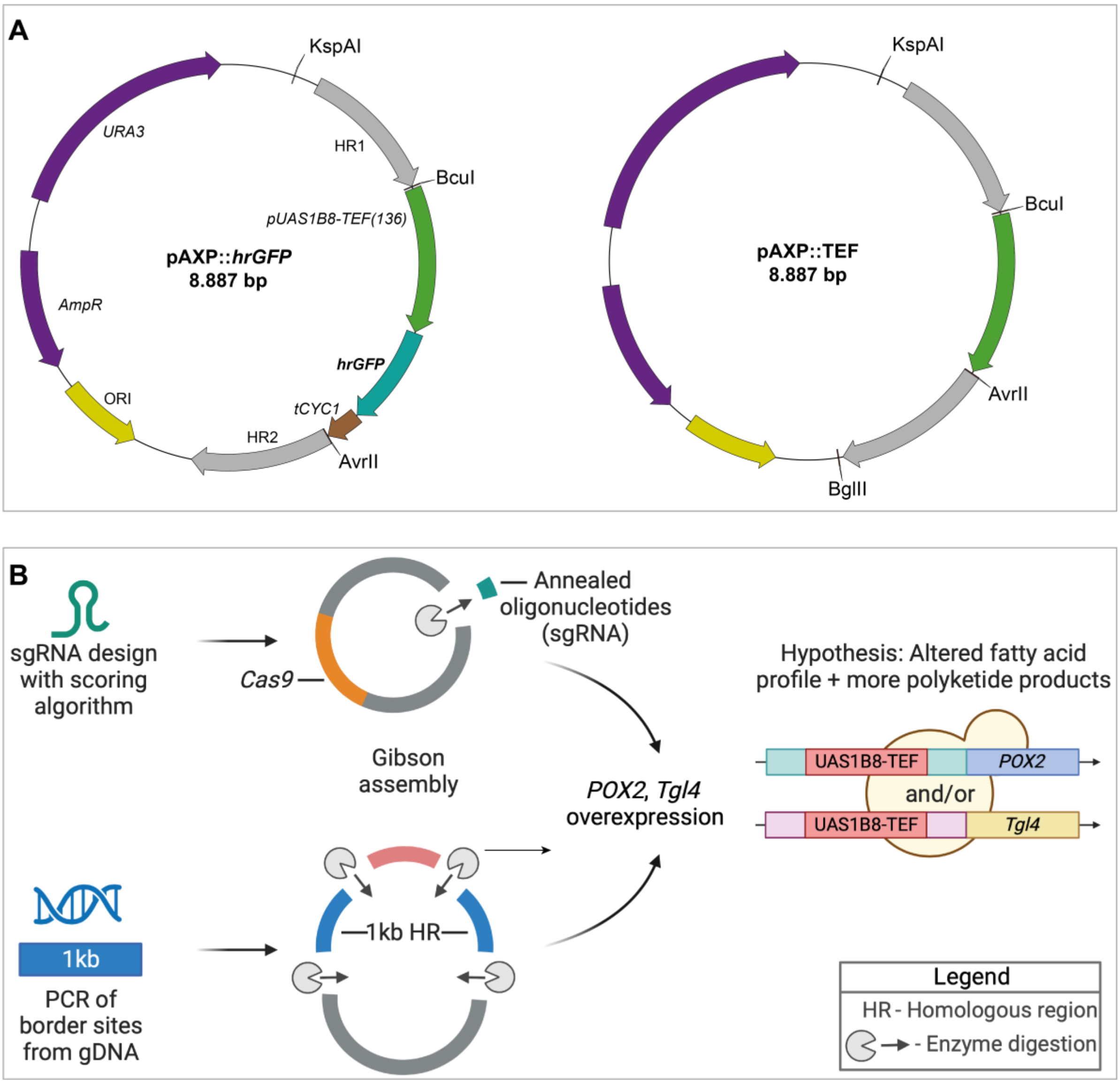

Supplementary Figure 2. Overexpression of β-oxidation pathway. **(A)** Addition of the BglII restriction site, downstream HR2, for facile removal and replacement of the homologous regions (HRs). **(B)** Generation of the pCRISPRyl plasmids containing the *Cas9* and sgRNA sequence, and in parallel, design of the corresponding 1kb donor homologous regions for the donor plasmid.

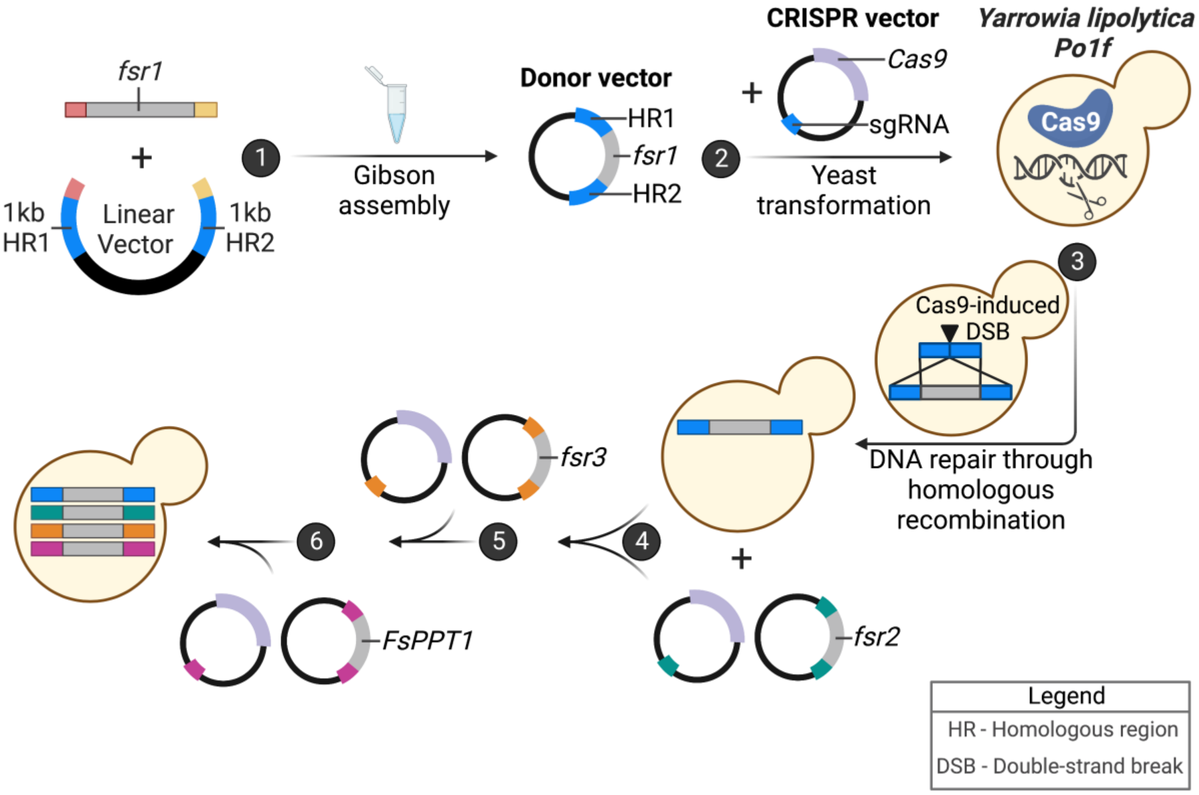

Supplementary Figure 3. Overview of the bostrycoidin biosynthetic pathway transfer process with the CRISPR-Cas9 system**.** Amplified genes and donor vector backbones were assembled via Gibson assembly using 30 bp overhangs. Subsequently, *Y. lipolytica* was transformed with the donor plasmid containing 1kb homologous regions up- and downstream of the integration cassette and the pCRISPRyl plasmid containing the *Cas9* gene and the corresponding sgRNA sequence (53). The genes were integrated iteratively after each transformation round.

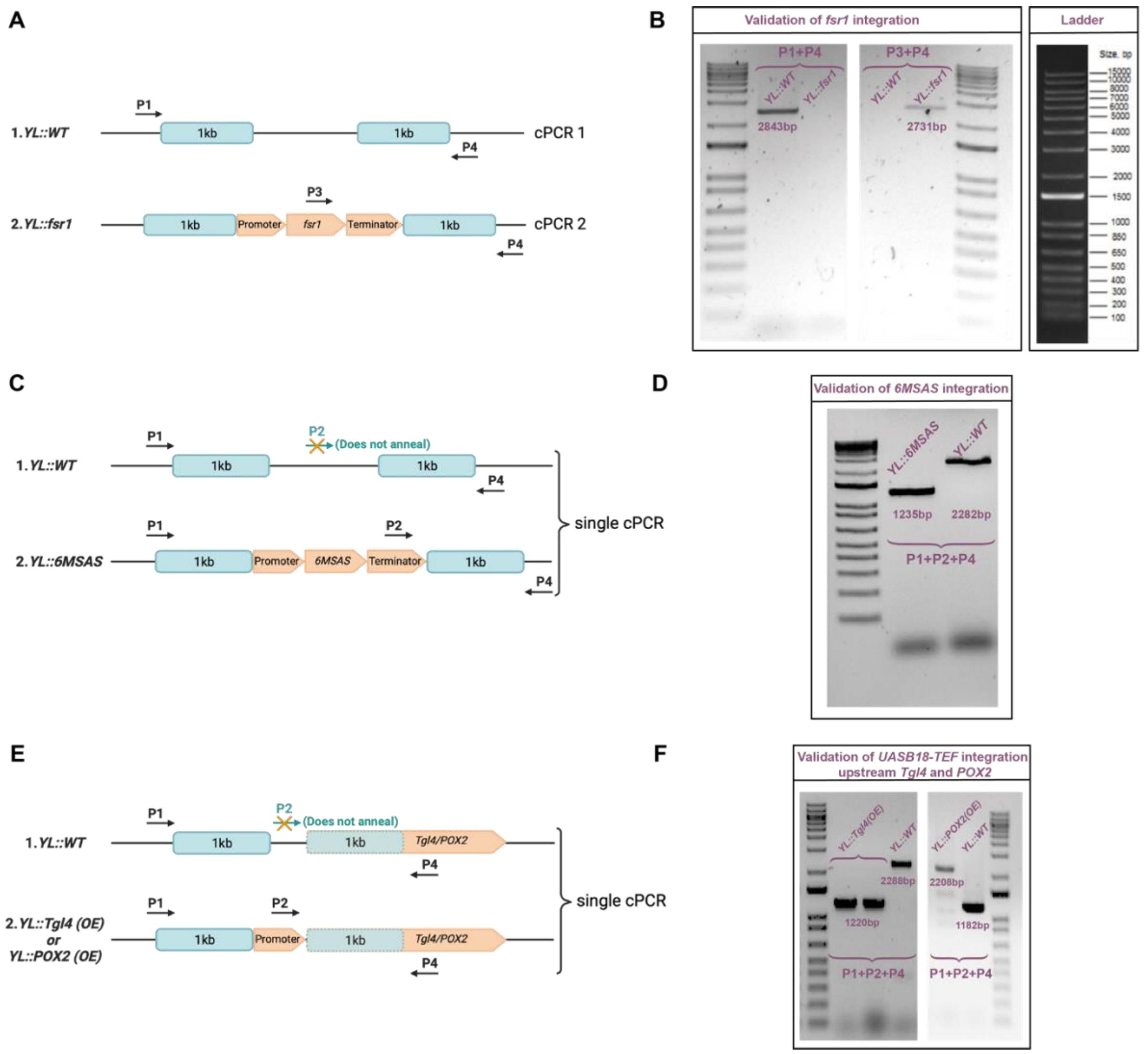

Supplementary Figure 4. Colony PCR-based (cPCR) transformant screening. **(A)** P1 and P4 amplify the wild type (WT) locus outside the HR, yielding a 2.8kb product, while P3 is a unique primer matching the *fsr1*, thus annealing into the gene and, together with P4, yielding a 2.7kb product when the cassette has been integrated; **(B)** cPCR results with the two-primer system for *fsr1* integration (Full scan of the original gel – **Supplementary Figure 6**). The elongation time in the PCR program was set only for amplification of a maximum of 3kb, rather than the entire integrated cassette, thus allowing for a quicker screening process; **(C)** P1 and P3 amplify the WT locus outside the HR, yielding a 2.3kb band. P2 is a general primer that anneals to the cassette in the terminator region and is thus added to the PCR mix with the other two primers, yielding a 1.2kb band, in case of successful cassette integration; **(D)** Example of cPCR results with the three-primer PCR system, based on *6MSAS* integration (Full scan of the original gel – **Supplementary Figure 7**). The elongation time was set for a fragment of a maximum of 2.5kb; **(E)** P1 and P3 amplify the region upstream and a part of *Tgl4/POX2*, yielding a 2.2kb band. P2 is a general primer that anneals to the TEF(136) promoter, yielding a 1.2kb band, in case of successful promoter integration upstream *Tgl4/POX2*; (**F**) Example of screening results for promoter integration (Full scans of the original gel – **Supplementary Figures 8, 9**). The elongation time was set for the elongation of a maximum of 2.5kb.

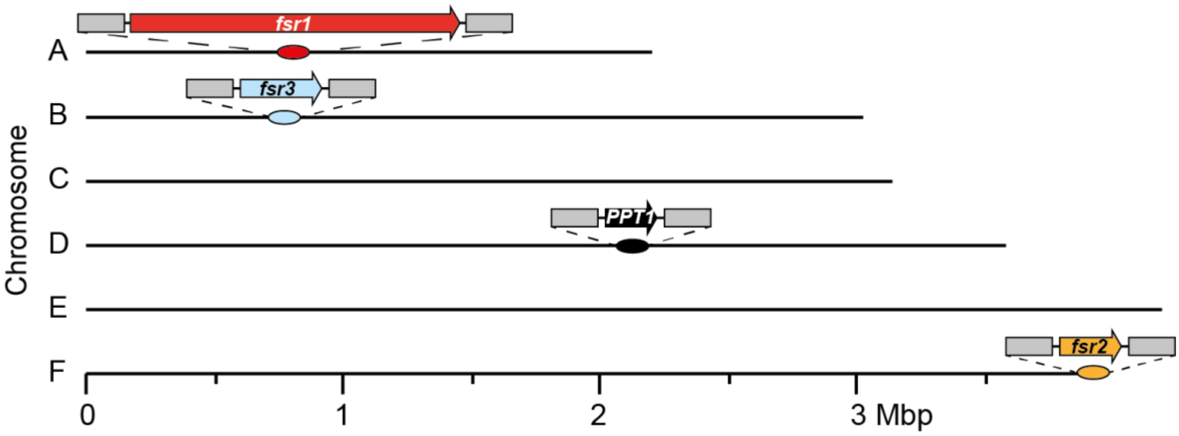

Supplementary Figure 5. Genomic integration of *FsPPT1*, *fsr1*, *fsr2*, and *fsr3* into four different loci of the *Y. lipolytica* Po1f genome.

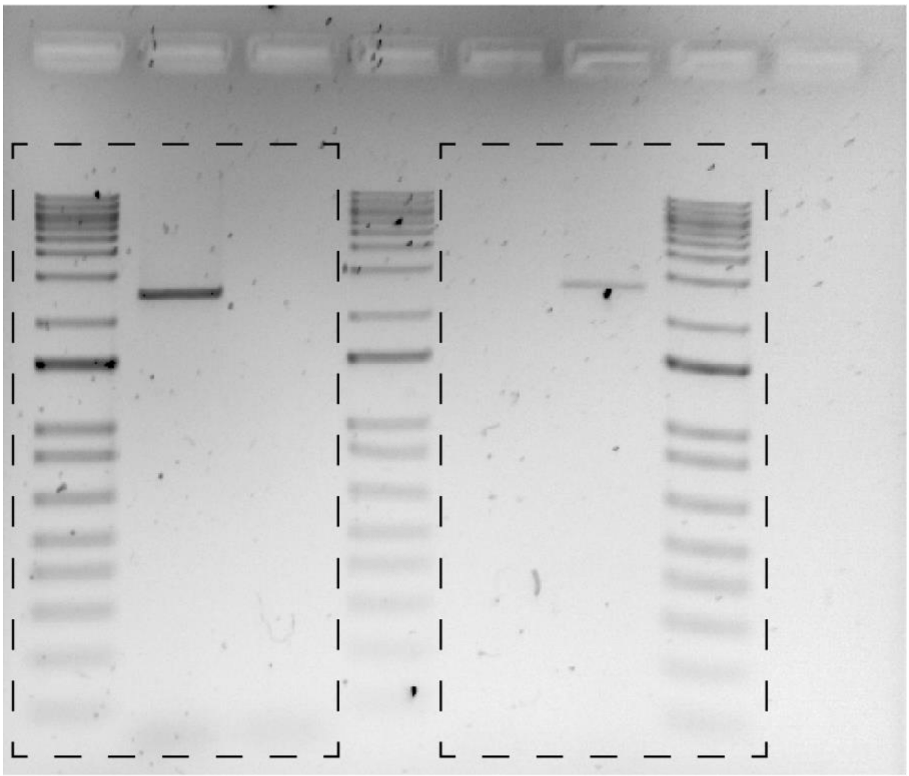

Supplementary Figure 6. Full scan of the original gel demonstrating the cPCR results with the two-primer system for *fsr1* integration. The areas marked with dashed rectangles are presented in **Supplementary Figure 4 B**.

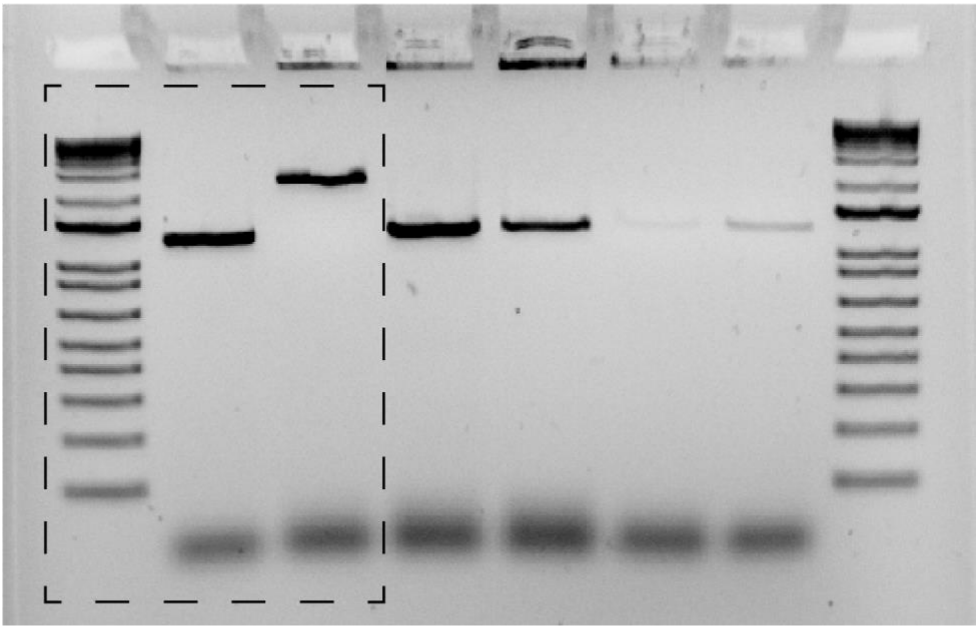

Supplementary Figure 7. Full scan of the original gel demonstrating the cPCR results with the three-primer system for *6MSAS* integration. The area marked with a dashed rectangle is presented in **Supplementary Figure 4 D**. The figure represents a transformation screening gel; the remainder of the wells not included in the figure from the main article indicate a successful integration of *6MSAS* in the genome and generation of several *YL::6MSAS* strains.

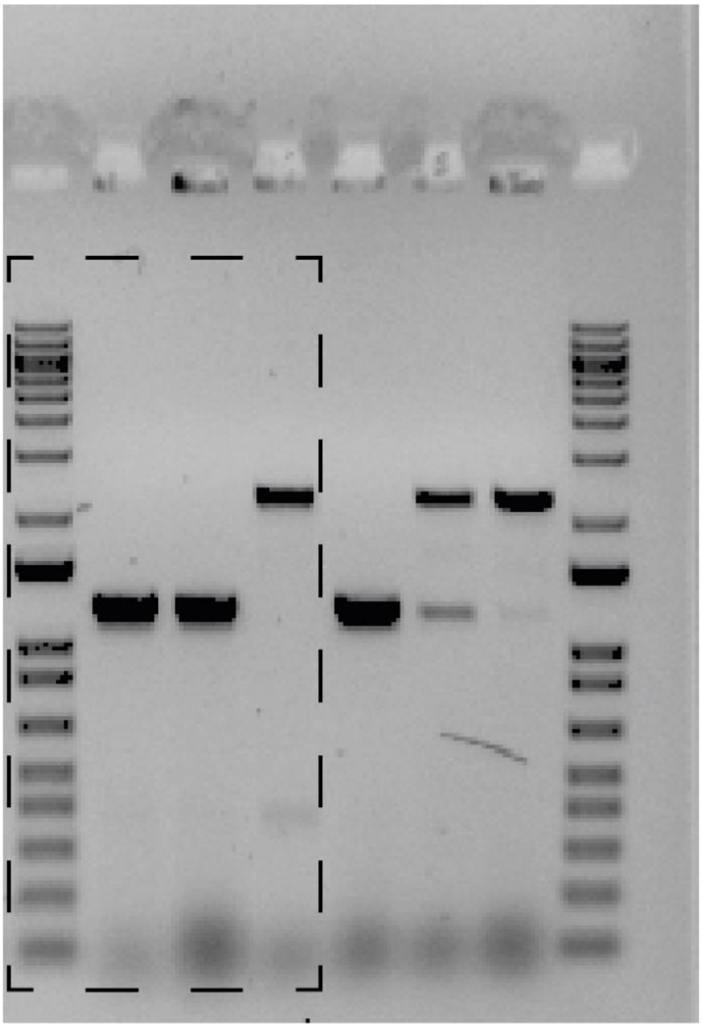

Supplementary Figure 8. Full scan of the original gel demonstrating the cPCR results with the three-primer system for UAS1B8-TEF(136) integration upstream *Tgl4*. The area marked with a dashed rectangle is presented in **Supplementary Figure 4 F**. The figure represents a transformation screening gel; the remainder of the wells not included in the figure from the main article indicate successful/unsuccessful integration of the promoter in the genome, depending on the band size.

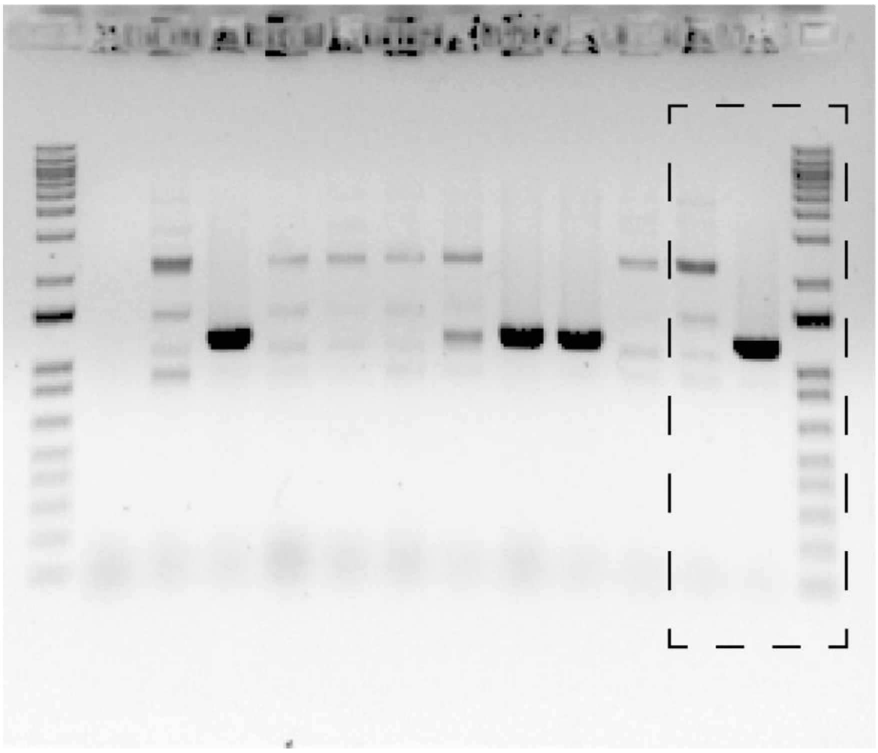

Supplementary Figure 9. Full scan of the original gel demonstrating the cPCR results with the three-primer system for UAS1B8-TEF(136) integration upstream *POX2*. The area marked with a dashed rectangle is presented in **Supplementary Figure 4 F**. The figure represents a transformation screening gel; the remainder of the wells not included in the figure from the main article indicate successful/unsuccessful integration of the promoter in the genome, depending on the band size.
